# Nkx2‐5 Is Expressed in Atherosclerotic Plaques and Attenuates Development of Atherosclerosis in Apolipoprotein E–Deficient Mice

**DOI:** 10.1161/JAHA.116.004440

**Published:** 2016-12-19

**Authors:** Meng Du, Xiaojing Wang, Xin Tan, Xiangrao Li, Dandan Huang, Kun Huang, Liu Yang, Fengxiao Zhang, Yan Wang, Dan Huang, Kai Huang

**Affiliations:** ^1^ Department of CardiologyUnion HospitalTongji Medical CollegeHuazhong University of Science and TechnologyWuhanChina; ^2^ Clinic Center of Human Gene ResearchUnion HospitalTongji Medical CollegeHuazhong University of Science and TechnologyWuhanChina

**Keywords:** atherosclerosis, matrix metalloproteinases, NK2 homeobox 5, plaque stability, Atherosclerosis, Animal Models of Human Disease, Gene Expression & Regulation

## Abstract

**Background:**

NK2 homeobox 5 (Nkx2‐5) is a cardiac homeobox transcription factor that is expressed in a broad range of cardiac sublineages. Embryos lacking Nkx2‐5 are nonviable attributed to growth retardation and gross abnormalities of the heart. However, the role of Nkx2‐5 in atherosclerosis remains elusive. This study aims to elucidate the specific functions of Nkx2‐5 during atherogenesis and in established atherosclerotic plaques.

**Methods and Results:**

Two types of atherosclerotic lesions were created in ApoE^−/−^ mice through 2 different dietary manipulations. Mice fed a standard chow diet were sacrificed at 20 weeks old, a time point at which mice developed early‐stage atherosclerotic lesions. The other half of mice were fed a western diet from 6 to 22 weeks old and then sacrificed. These mice demonstrated advanced atherosclerosis. No Nkx2‐5 was detected in normal arteries; however, it was abundantly present in the intima of atherosclerotic lesions and localized in macrophages and smooth muscle cells. Adenovirus gene transfer of Nkx2‐5 markedly ameliorated and stabilized the atherosclerotic plaques, and knockdown of Nkx2‐5 significantly exacerbated the disease. Molecular studies indicated that expression of specific members of matrix metalloproteinases and tissue inhibitor of metalloproteinases, which play a crucial role in the progression of atherosclerosis, were directly regulated by Nkx2‐5. Furthermore, we demonstrated that the compromised endothelial function, which was considered as a hallmark of early atherosclerosis, could be improved by Nkx2‐5 gene transfer.

**Conclusions:**

Nkx2‐5 exerts antiatherogenic effects, which may partly be attributed to regulation on matrix metalloproteinases and tissue inhibitor of metalloproteinases, thus stabilizing atherosclerotic plaque; besides, it improves endothelial function by inhibiting leukocyte adhesion to the endothelium.

## Introduction

NK2 homeobox 5 (Nkx2‐5) is a cardiac homeobox transcription factor that is expressed in a broad range of cardiac sublineages, from the early committed cardiac progenitors through the adult cardiomyocytes, and plays a pivotal role in the regulation of cardiac, vascular, and hematopoietic lineages.^1^, ^2^, ^3^, ^4^, ^5^, ^6^, ^7^ The human gene, NKX2‐5, is, to date, the most commonly mutated single gene in congenital heart disease, accounting for 1% to 4% of specific malformations, including atrial septal defect, double‐outlet right ventricle, and a complex congenital condition arising from stenosis of the pulmonary artery termed tetralogy of Fallot.^8^, ^9^, ^10^, ^11^ Studies in mice show that Nkx2‐5 is required for specification and spatial definition of chamber myocardium and for formation and maintenance of elements of the conduction system.^4^, ^5^, ^12^, ^13^, ^14^

Whereas the majority of research on Nkx2‐5 is focused on its role in heart formation, recent studies have provided new insight into certain relevance of Nkx2‐5 to many other pathophysiological conditions, such as early‐onset and severe preeclampsia, thyroid dysgenesis, keratinocyte differentiation, and acute T‐lymphoblastic leukemia.^15^, ^16^, ^17^, ^18^, ^19^, ^20^ These studies, although limited, unveiled a novel role of Nkx2‐5 as a broad‐acting regulatory component beyond cardiogenesis.

In the present study, we showed that expression of Nkx2‐5 was increased in atherosclerotic lesions of humans and ApoE^−/−^ mice. Nkx2‐5 gene transfer markedly ameliorated atherosclerosis, with a diminished lesion area and a more‐stable plaque phenotype, and knockdown of Nkx2‐5 significantly exacerbated the disease. Then, we investigated the mechanisms whereby Nkx2‐5 protects against atherosclerosis through its multiple actions in macrophages, smooth muscle cells, and endothelial cells.

## Material and Methods

### Human Atherosclerotic Tissues

Twelve human atherosclerotic lesions were collected from patients undergoing carotid endarterectomy at Wuhan Union Hospital. Ten internal mammary arteries and 14 saphenous veins obtained from patients undergoing coronary artery bypass surgery were used as nonatherosclerotic control arteries. Written informed consent was obtained from all participants according to the declaration of Helsinki. The investigations were approved by the Ethical Committee of Huazhong University of Science and Technology.

### Immunofluorescence

Immunofluorescence on human carotid artery sections and mice aortic sinus sections was performed as described.^21^, ^22^ Briefly, for double staining, sections were incubated with mouse anti‐Nkx2‐5 antibody (sc‐376565X; Santa Cruz Biotechnology, Santa Cruz, CA), together with rabbit anti‐F4/80 antibody (ab100790; Abcam, Cambridge, MA), anti‐α‐SMA (alpha‐smooth muscle actin) antibody (ab124964; Abcam), or anti–von Willebrand factor (vWF) antibody (ab9378; Abcam) at 4°C overnight, followed by a 30‐minute incubation with secondary antibody conjugated to Alexa Fluor 568 (red) and Alexa Fluor 488 (green) (Molecular Probes, Inc., Eugene, OR). Signals of individual and merged images for antigen detection were performed using a fluorescence microscope (Olympus, Tokyo, Japan) and AxioVision (version 4.8; Carl Zeiss GmbH, Jena, Germany) software.

### Generation of Recombinant Adenovirus

Replication‐defective recombinant adenovirus carrying the entire coding sequence of Nkx2‐5 (Ad‐Nkx2‐5) was constructed with the Adenovirus Expression Vector Kit (Takara Bio Inc., Kusatsu, Japan). An adenovirus‐only–containing green fluorescence protein (GFP) was used as a negative control (Ad‐EV). To generate adenovirus expressing shRNA against Nkx2‐5 (Ad‐shNkx2‐5), 3 siRNAs for mouse Nkx2‐5 were designed and the one with the optimal knockdown efficiency was chosen to create shRNA and then recombined into adenoviral vectors. The target sequence is as follows: GCTTCAAGCAACAGCGGTA. The negative control adenovirus was designed to express nontargeting “universal control” shRNA (Ad‐shNC). Amplification and purification of recombinant adenovirus was performed according to the manufacturer's instructions (Takara Bio).

### Animals

Six‐week‐old male C57BL/6 ApoE^−/−^ mice were bred and maintained under conventional housing conditions in our animal facility. These mice were divided into 8 groups (n=16 for every group). Half of them were fed a standard chow diet until sacrificed at 20 weeks old, a time point at which mice developed early‐stage atherosclerotic lesions. The other 4 groups were fed a Western diet from 6 to 22 weeks old, and then the mice were sacrificed. These mice demonstrated advanced atherosclerosis. Four weeks before euthanization, animals were anesthetized with pentobarbital sodium (50 mg/kg, intraperitoneally, P3761; Sigma‐Aldrich, St. Louis, MO), and either Ad‐EV, Ad‐Nkx2‐5, Ad‐shNC, or Ad‐shNkx2‐5 (5×10^9^ plaque‐forming units/mouse) was injected directly into the left ventricle of mice from each group fed chow diet or Western diet, respectively. Handling of animals was in accord with institutional guidelines.

### Histological Analysis and Quantification of Atherosclerotic Lesions

Mice were fasted for 4 hours and then anesthetized. Atherosclerosis assays were performed at the indicated times of sacrifice. Lesion areas of thoracoabdominal aorta were determined by en face Oil Red O staining as described.^23^ Aortas were dissected longitudinally with an extremely fine Vanna microscissor and pinned flat on a black wax surface with 0.2‐mm‐diameter stainless steel pins. Pinned aortas were stained with Oil Red O, and images were captured with a standard digital camera. For microscopic evaluation of the aortic sinus lesions, hearts were fixed in 4% PFA and cryopreserved in 15% sucrose and then 30% sucrose. After being embedded in optimum cutting temperature compound (Sakura Finetek, Torrance, CA), hearts were cryosectioned and 6‐μm sections were collected at 80‐μm intervals, starting at a 100‐μm distance from the appearance of the aortic valves. Aortic sinus sections were stained for lesion areas with Oil Red O, morphology with hematoxylin and eosin (HE), collagen content with Masson's trichrome staining (Masson), and elastic fibers with elastica van Gieson staining (EVG). The relative content of macrophages, smooth muscle cells, adhesion molecules, matrix metalloproteinases (MMPs), and tissue inhibitor of metalloproteinases (TIMPs) were detected by immunohistochemistry. Frozen serial sections were treated with 0.3% H_2_O_2_ in PBS to block endogenous peroxidase activity, followed by blocking in 4% BSA (Sigma‐Aldrich). Primary antibodies were specific for the following: F4/80 (ab100790; Abcam); α‐SMA (ab124964; Abcam); vWF (ab9378; Abcam); MMP2 (AP51352; Abgent); MMP9 (AP6214a; Abgent, San Diego, CA); MMP3 (AP51354 Abgent), MMP8 (AP51356; Abgent); MMP14 (AP6198a; Abgent); TIMP1 (ab86482, Abcam), E‐selectin (ab18981; Abcam); and vascular cell adhesion molecule‐1 (VCAM1; ab134047; Abcam). All sections were stained with biotinylated secondary antibodies and detected using ABC reagents (Vector Laboratories, Burlingame, CA). Collected images were quantitated by quantitative morphometry using the Image Pro Plus program. Lesion areas were determined by calculating the mean lesion area of the 4 sections at 80‐μm intervals. For evaluation of the relative content of the stained constituents, we determined the percentage of blue (collagen on Masson's trichrome staining) and diaminobenzidine‐positive (immunohistochemistry) areas to the total plaque areas. Fibrous caps were defined as the vascular smooth muscle cell (VSMC)‐ and collagen‐rich area overlying the cholesterol‐rich, matrix‐poor, and acellular regions of the necrotic cores. In EVG‐stained sections, the number of ruptures (ie, discontinuities or fractures) of the elastic lamina was determined.

### In Situ Zymography

To detect and localize gelatinolytic activity of MMPs in aortic sinus, in situ zymography was carried out as described previously.^24^ Briefly, 100 μg/mL of fluorescein‐conjugated dye‐quenched (DQ) gelatin (Molecular Probes) was mixed with 0.2% agarose melted in reaction buffer (50 mmol/L of Tris‐HCl [pH 7.5] 0.15 mol/L of NaCl, 5 mmol/L of CaCl_2_, and 0.2 mmol/L of sodium azide). Aortic sinus sections were incubated with the reaction mixture prepared above for 24 hours at 37°C in a moist dark chamber and then rinsed 3 times in PBS and mounted in Vectashield mounting medium (Vector Laboratories) for fluorescence microscopy. When DQ gelatin is cleaved by gelatinolytic activity, fluorescent peptides are released that are visible against a weakly fluorescent background.

### Cell Culture

Peripheral blood mononuclear cells (PBMCs) were prepared on a Ficoll‐Hypaque density gradient centrifugation. CD14^+^ cells were obtained through positive selection by CD14^+^ micromagnetic beads according to the manufacturer's instructions (Miltenyi Biotec, Bergisch Gladbach, Germany). Then, monocytes were differentiated in complete RPMI‐1640 medium supplemented with recombinant human macrophage colony‐stimulating factor (50 ng/mL; R&D Systems, Minneapolis, MN) for an additional 6 days before following study. Human primary aortic smooth muscle cells (HASMCs; ATCC PCS‐100‐012; American Type Culture Collection [ATCC], Manassas, VA) and human primary aortic endothelial cells (HAECs; ATCC PCS‐100‐011; ATCC) were cultured according to the manufacturer's instructions. Cells were used for experiments at passages 3 to 8.

### Western Blot

Cells or tissues were homogenized in ice‐cold suspension buffer supplemented with a proteinase inhibitor cocktail (Sigma‐Aldrich) as described previously.^25^ Protein concentrations were determined using the BCA Protein assay kit (Thermo Scientific, Waltham, MA). Equal amounts of protein were fractionated by SDS polyacrylamide gels, followed by immunoblotting with the following primary antibodies: MMP1 (AP51345; Abgent); MMP2 (AP51352; Abgent); MMP3 (AP51354; Abgent); MMP7 (ALS11637; Abgent); MMP8 (AP51356; Abgent); MMP9 (AP6214a; Abgent); MMP10 (AP50649; Abgent); MMP11 (AP51347; Abgent); MMP12 (AP6196a; Abgent); MMP13 (AP51348; Abgent); MMP14 (AP6198a; Abgent); TIMP1 (sc‐21734; Santa Cruz Biotechnology); TIMP2 (sc‐365671; Santa Cruz Biotechnology); TIMP3 (sc‐373839; Santa Cruz Biotechnology); TIMP4 (sc‐9375; Santa Cruz Biotechnology); intracellular adhesion molecule‐1 (ICAM1; ab124759; Abcam); VCAM1 (ab134047; Abcam), E‐selectin (ab18981; Abcam), P‐selectin (ab178424; Abcam); nuclear factor kappa B (NF‐κB) Pathway Sampler Kit (9936; Cell Signaling Technology [CST], Danvers, MA); and phospho‐MAPK (mitogen‐activated protein kinase) Family Antibody Sampler Kit (9910; CST). Membranes were then incubated with peroxidase‐conjugated secondary antibody, and specific bands were detected with a Bio‐Rad (Hercules, CA) imaging system.

### Real‐Time Reverse‐Transcriptase Polymerase Chain Reaction

Total RNA was extracted from cells with the use of TRIzol reagent (D9108A; Takara Bio). RNA was reverse‐transcribed using the RNA PCR Kit (RR036A; Takara Bio). Quantitative polymerase chain reaction (PCR) amplification was performed with an ABI PRISM 7900 Sequence Detector system (Applied Biosystem, Foster City, CA), according to the manufacturer's instructions. The real‐time PCR primer sequences are shown in Table S1.

### Adhesion Assay

Adhesion assay was performed using Endothelial Cell Adhesion Assay Kit (ECM645; Bioscience Research Reagents [formerly Chemicon], Billerica, MA) according to the kit instructions, with modifications. HAECs (PCS‐100‐011; ATCC) were grown on chamber slides where adhesion assay was performed. Cells were infected with Ad‐Nkx2‐5 or Ad‐EV, followed by treating with tumor necrosis factor alpha (TNFα; 10 ng/mL) for 6 hours. Fresh adult human PBMCs (PCS‐800‐011; ATCC) were labeled with calcein acetomethoxy (AM) dye: PBMCs were pelleted at 240*g* for 10 minutes, resuspended in 1 mL of culture medium with 2.5 μmol/L of calcein AM from the kit, and incubated at 37°C (5% CO_2_) for 30 minutes. PBMCs were then washed 3 times with HAEC media and added to HAEC cells (150 000 labeled PBMCs per chamber). PBMCs were allowed to settle down and adhere to HAECs for 1 hour. Then, unbound PBMCs were washed out by gentle removal and addition of culture medium in chambers 3 times. Following the washing of unbound PBMCs, HAECs with attached PBMCs were fixed for 10 minutes in 2% formaldehyde at room temperature, permeabilyzed with 0.1% Triton X‐100, stained with 2.5 μg/mL of 4′,6‐diamidino‐2‐phenylindole (DAPI; DNA stain; Invitrogen, Carlsbad, CA) and mounted with ProLong Gold antifade reagent (Invitrogen). PBMCs were identified and counted based on calcein AM green fluorescence (excitation, 495 nm; emission, 516 nm), and total number of cells was counted based on DAPI fluorescence (350‐nm excitation, 470‐nm emission).

### Luciferase Assays

Promoters of members of MMPs and TIMPs, E‐selectin, P‐selectin, VCAM1, and ICAM1 were subcloned into the pGL3‐Basic vector (Promega, Madison, WI) using the One Step Cloning Kit (C112‐02; Vazyme Biotech Ltd., Nanjing, China). Primers and the length of PCR products (the restriction enzyme sites are not included) are demonstrated in Table S2. Luciferase reporter constructs were cotransfected with an internal control plasmid, pRL‐TK (Renilla luciferase reporter plasmid; Promega), into HEK293 cells, followed by infection with Ad‐Nkx2‐5 or Ad‐EV. Then, cells were harvested, lysed, and the luciferase activity was determined with the Dual Luciferase Reporter Assay Kit (Promega), according to the manufacturer's instruction.

### Chromatin Immunoprecipitation Assay

Chromatin immunoprecipitation (ChIP) assay was performed according to instructions (CHIP assay kit; Millipore, Billerica, MA) using mouse monoclonal antibodies against Nkx2‐5 (sc‐376565X; Santa Cruz Biotechnology). DNA samples recovered after immunoprecipitation were subjected to PCR using primers demonstrated in Table S3. As negative controls, ChIP was performed in the presence of mouse immunoglobulin G (IgG).

### Statistical Analysis

GraphPad Prism software (GraphPad Software Inc., La Jolla, CA) was used for statistical analyses. Data were normally distributed, as assessed by using the Kolmogorov–Smirnov test. Comparisons between groups were carried out using the Student *t* test. Data are presented as mean±SEM, and a value of *P*<0.05 was considered as statistically significant.

## Results

### Nkx2‐5 Is Expressed in Human and Mice Atherosclerotic Plaque

**Figure 1:**
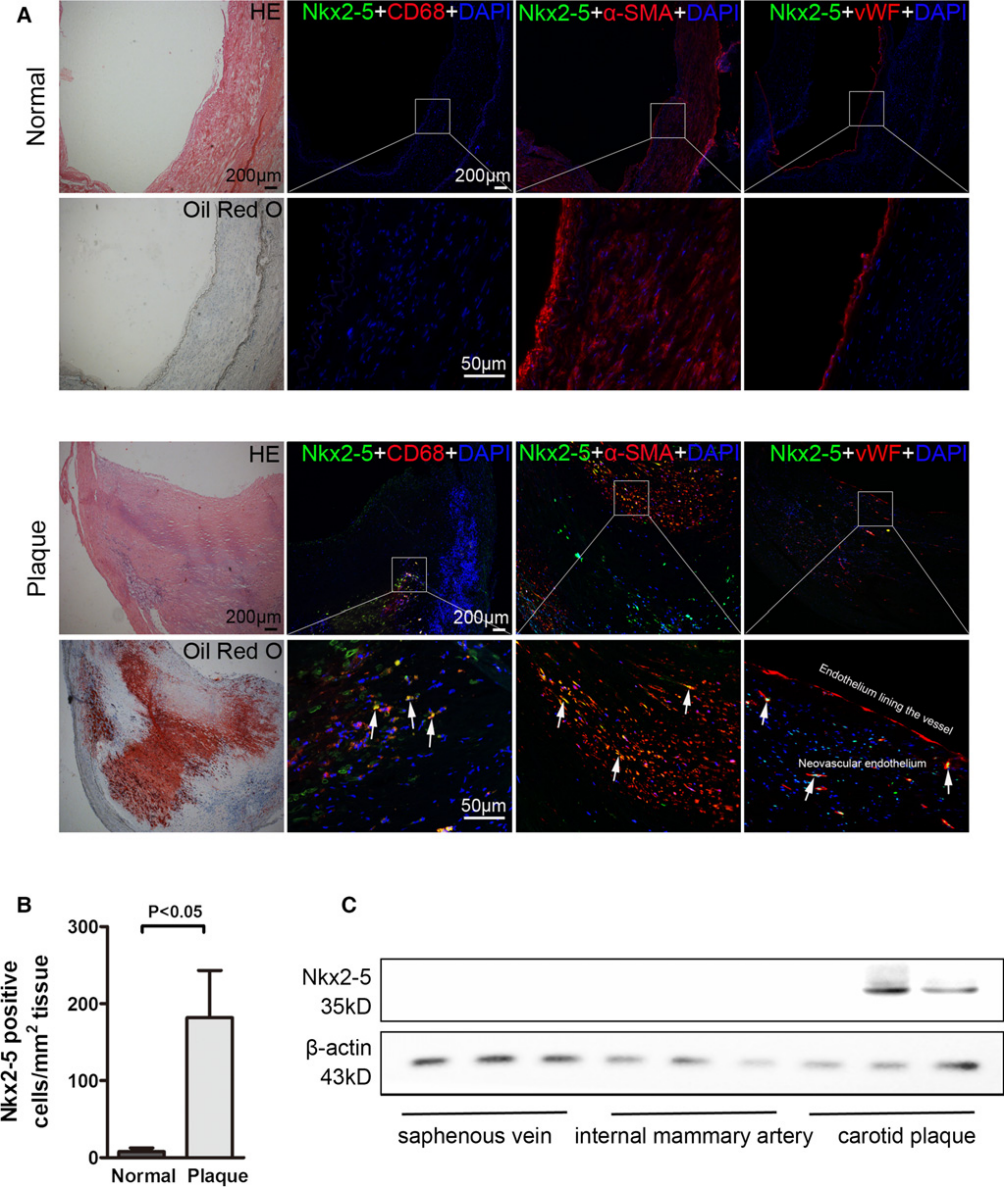
NK2 homeobox 5 (Nkx2‐5) expression in human normal vessels and atherosclerotic plaques. A, Immunofluorescence assay of Nkx2‐5 in internal mammary arteries (normal, upper) and atherosclerotic lesions of carotid arteries (plaque, lower). For colocalization analysis, sections were costained for Nkx2‐5 (green) and CD68 (red, macrophage marker), α‐SMA (red, smooth muscle cell marker), or von Willebrand Factor (vWF; red, endothelial marker). 4′,6‐diamidino‐2‐phenylindole (DAPI) was used for nucleus staining (blue). Arrows indicate Nkx2‐5 and cell‐specific marker double‐positive cells. B, Quantitative analysis of Nkx2‐5‐positive cells in (A). n=5 for each group. C, Western blot for Nkx2‐5 expression in human control vessels (saphenous vein and internal mammary artery) and carotid atherosclerotic plaques. n=12 for atherosclerotic plaques, 10 for internal mammary artery, and 14 for saphenous vein. HE indicates hematoxylin and eosin; α‐SMA, alpha‐smooth muscle actin.

To characterize the involvement of Nkx2‐5 in atherosclerosis, we first examined expression of Nkx2‐5 in several human healthy vessels (saphenous vein and internal mammary artery) and carotid atherosclerotic plaques. As double immunofluorescent staining demonstrated, Nkx2‐5 was virtually undetectable in healthy arteries. However, in atherosclerotic plaque, Nkx2‐5 was expressed predominantly in macrophages and smooth muscle cells that exhibited positive staining for CD68 or α‐SMA, respectively. Little‐to‐no Nkx2‐5 immunoreactivity was detected in medial smooth muscle cells beneath plaque or endothelial cells lining the lumen of the diseased vessel. But, surprisingly, a certain number of Nkx2‐5 and vWF double‐positive cells could be observed in the subendothelial neovascular (Figure 1A and 1B). Moreover, Nkx2‐5 protein levels, as detected by western blot, were markedly increased in plaques (2 of 3 plaques contained Nkx2‐5) versus control vessels (Figure 1C). Given that ApoE^−/−^ mice demonstrate the atherosclerosis phenotype, we further performed immunostaining to analyze expression of Nkx2‐5 in diseased ApoE^−/−^ mice and normal C57BL/6 mice. Consistent with our previous findings in human atherosclerotic plaque, evident immunoreactivity for Nkx2‐5 was detected in macrophages and smooth muscle cells in aortic sinus lesions of ApoE^−/−^ mice, whereas normal C57BL/6 mice demonstrated no positive staining for Nkx2‐5 (Figure S1A). Western blot was further conducted to determine the protein levels of Nkx2‐5 in mice. As demonstrated in Figure S1B, Nkx2‐5 protein was relatively abundant in heart, liver, lung, kidney, and adipose tissue, whereas it was barely detectable in spleen, aorta, and muscle. As expected, Nkx2‐5 levels in aorta of ApoE^−/−^ mice were increased with age, and more markedly when mice were fed with a Western diet. However, we failed to detect Nkx2‐5 expression in aorta of C57BL/6 mice, even on the Western diet (Figure S1C). These results indicated that upregulation of Nkx2‐5 was associated with severity of atherosclerosis rather than age or diet.

### Evaluation of Adenovirus Infection Efficiency in Arterial Walls of Experimental Atherosclerotic Mice After Intraventricular Administration

To identify the role of Nkx2‐5 in atherosclerosis, recombinant adenovirus were generated and injected directly into left ventricles of anesthetized animals by heart puncture. To evaluate the efficiency of gene transfer in aortic tissue of animals, expression of GFP in various tissues was examined by western blot and compared with that of tail vein injection. As illustrated in Figure S2A, GFP protein was detected mainly in liver and aortic tissue of mice receiving adenovirus intraventricularly for 1 week. However, adenovirus infection efficiency in aorta was relatively low when administrated through the tail vein. Time‐course experiments revealed that transgene expression in aorta was evident at 1 week and declined progressively at 2 and 4 weeks after virus administration (Figure S2B). Considering the spatial distribution and temporal variation of the transgene expression, adenovirus was administrated intraventricularly 4 weeks before mice were sacrificed, as depicted in Figure S2C. To further confirm infection efficiency, Nkx2‐5 expression of aorta was examined by western blot. Whether fed a standard chow diet or a Western diet, further increase in Nkx2‐5 protein was detected in aortic tissue of mice receiving Ad‐Nkx2‐5, which was sustained until experiment termination. Conversely, no Nkx2‐5 protein was detectable in aorta from mice injected with Ad‐shNkx2‐5 (Figure S2D).

### Effect of Nkx2‐5 on Initiation and Progression of Atherosclerosis

**Figure 2:**
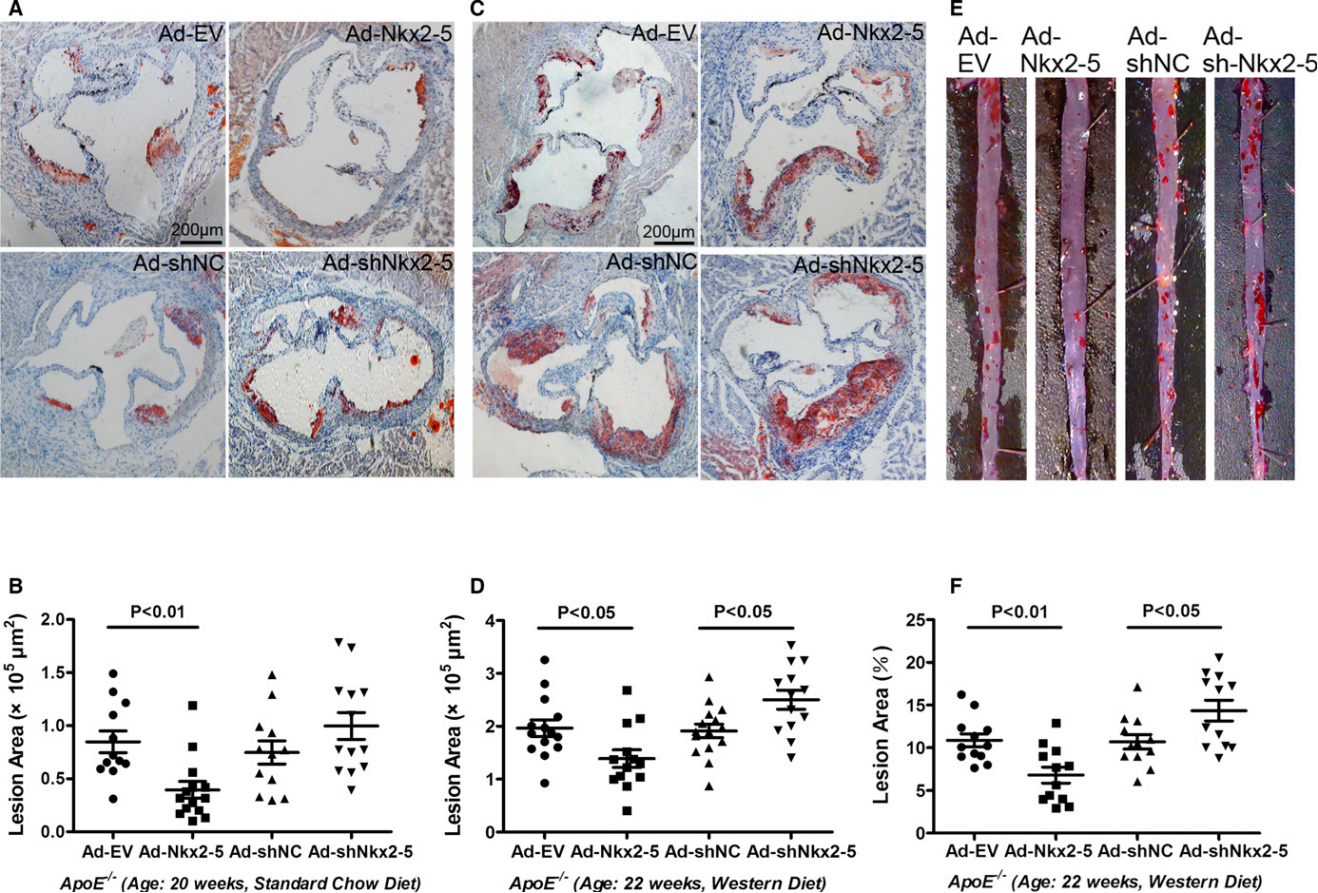
NK2 homeobox 5 (Nkx2‐5) exerts protective effect in the initiation and progression of atherosclerosis in ApoE^−/−^ mice. A and B, ApoE^−/−^ mice fed a chow diet were administrated adenovirus and sacrificed at 20 weeks. Cross‐sections of aortic sinus were stained with Oil Red O, and lesion areas were quantified. Data are expressed as mean±SEM (n=12 for Ad‐EV, 14 for Ad‐Nkx2‐5, 12 for Ad‐shNC, and 13 for the Ad‐shNkx2‐5–treated group). C and D, ApoE^−/−^ mice fed a Western diet were administrated adenovirus and sacrificed at 22 weeks. Cross‐sections of aortic sinus were stained with Oil Red O, and lesion areas were quantified. Data are expressed as mean±SEM (n=14 for Ad‐EV, 13 for Ad‐Nkx2‐5, 15 for Ad‐shNC, and 13 for the Ad‐shNkx2‐5–treated group). E and F, ApoE^−/−^ mice fed a Western diet were administrated adenovirus and sacrificed at 22 weeks. Lesion areas of thoracoabdominal aorta were evaluated by en face staining with Oil Red O. Data are expressed as mean±SEM (n=12 per group).

Two types of atherosclerotic lesions (referred to as early and advanced stage) were created in ApoE^−/−^ mice through 2 different dietary manipulations as previously described (Figure S2C). Four weeks before euthanization, either Ad‐EV, Ad‐Nkx2‐5, Ad‐shNC, or Ad‐shNkx2‐5 was delivered by intraventricular injection. Body weights and plasma lipid profiles were unaffected by Nkx2‐5 gene transfer (Table S4). Proportions of CD3^+^, CD4^+^_,_ or CD8^+^ lymphocytes in spleen of animals fed a Western diet were not affected by Nkx2‐5. Neither did the relative mRNA expression of certain transcription factors representative for T‐cell subsets, including T‐bet, GATA3, RORc, and Foxp3 (Figure S3). The lesion area that developed at the aortic sinus of Ad‐Nkx2‐5‐treated mice fed a chow diet was decreased by 50.6%, compared to those of control littermates treated with Ad‐EV, whereas the difference in lesion areas was not statistically significant between Ad‐shNkx2‐‐ treated mice and Ad‐shNC‐treated mice (Figure 2A and 2B). For the Western diet–fed mice, which exhibited advanced lesions, the atherosclerotic lesion area in Ad‐Nkx2‐5‐treated mice, as determined by Oil Red O staining of the thoracoabdominal aorta, was significantly decreased by 45.2% when compared to Ad‐EV‐treated mice, and Nkx2‐5 knockdown obviously accelerated atherosclerotic plaque formation (increased by 40.3% compared to Ad‐shNC‐treated mice; Figure 2E and 2F). Additionally, histological evaluation showed that plaque areas in the aortic sinus were consistent with the gross observation (Figure 2C and 2D).

### Effects of Nkx2‐5 on Lesional Morphology of Advanced Atherosclerotic Plaque

**Figure 3:**
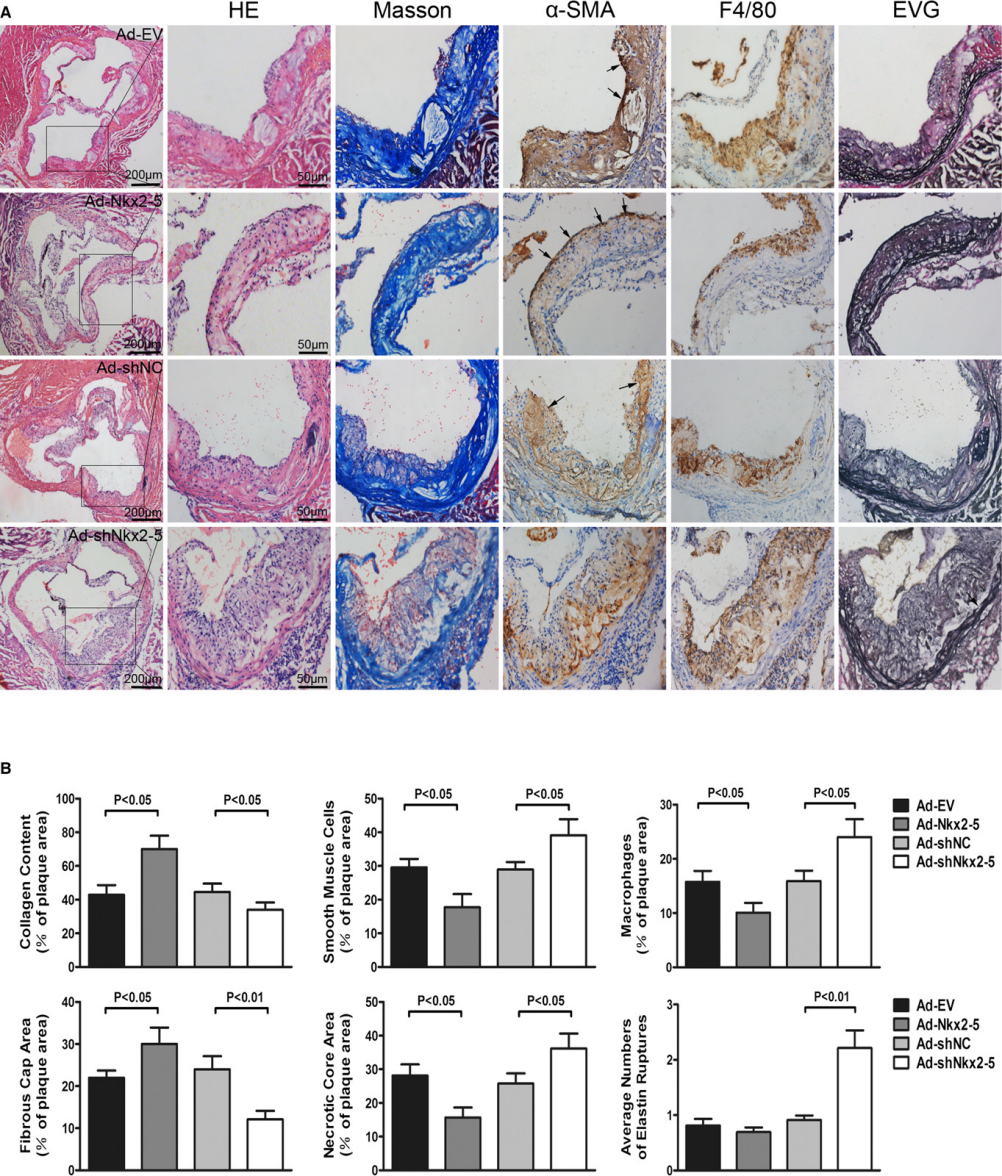
Histological analysis of aortic sinus lesions of ApoE^−/−^ mice fed a Western diet. A, Images show representative sections of aortic sinus from each group of mice stained with hematoxylin and eosin (HE), Masson's trichrome (Masson), elastica van Gieson (EVG), anti‐α‐SMA antibody, and anti‐F4/80 antibody. B, Collagen content, smooth muscle cells, macrophages, fibrous cap area, and necrotic core area are quantified as a percent of total plaque area. Elastic lamina destruction of the tunica media was evaluated by quantifying the ruptures of elastic fibers. Arrows in α‐SMA staining panels indicate smooth muscle cells favoring fibrous caps formation, and arrows in EVG staining panels indicate ruptures of elastic fibers of tunica media. Data are expressed as mean±SEM (n=12 per group). α‐SMA indicates alpha‐smooth muscle actin.

Nkx2‐5 not only affected plaque size, but also induced marked changes in morphology in ApoE^−/−^ mice fed a Western diet (Figure 3). Compared with Ad‐EV‐treated mice, plaques of Ad‐Nkx2‐5‐treated mice contained a relatively integral VSMC‐rich fibrous cap with abundant collagen and matrix overlying small necrotic cores. Macrophage content, determined by F4/80 immunostaining, was considerably decreased and degradation of elastic fibers in tunica media could hardly be observed. However, fibrous caps were markedly thinner and collagen content was decreased in Ad‐shNkx2‐5‐treated mice compared to their control littermates treated with Ad‐shNC. Although migratory smooth muscle cells significantly increased in Ad‐shNkx2‐5‐treated mice, they scattered throughout the lesions and tended to contribute to plaque expansion, instead of favoring fibrous cap formation. Besides, the infiltrating of macrophages and destruction of the elastic laminae were more pronounced in Ad‐shNkx2‐5‐treated mice compared to Ad‐shNC treated mice.

**Figure 4:**
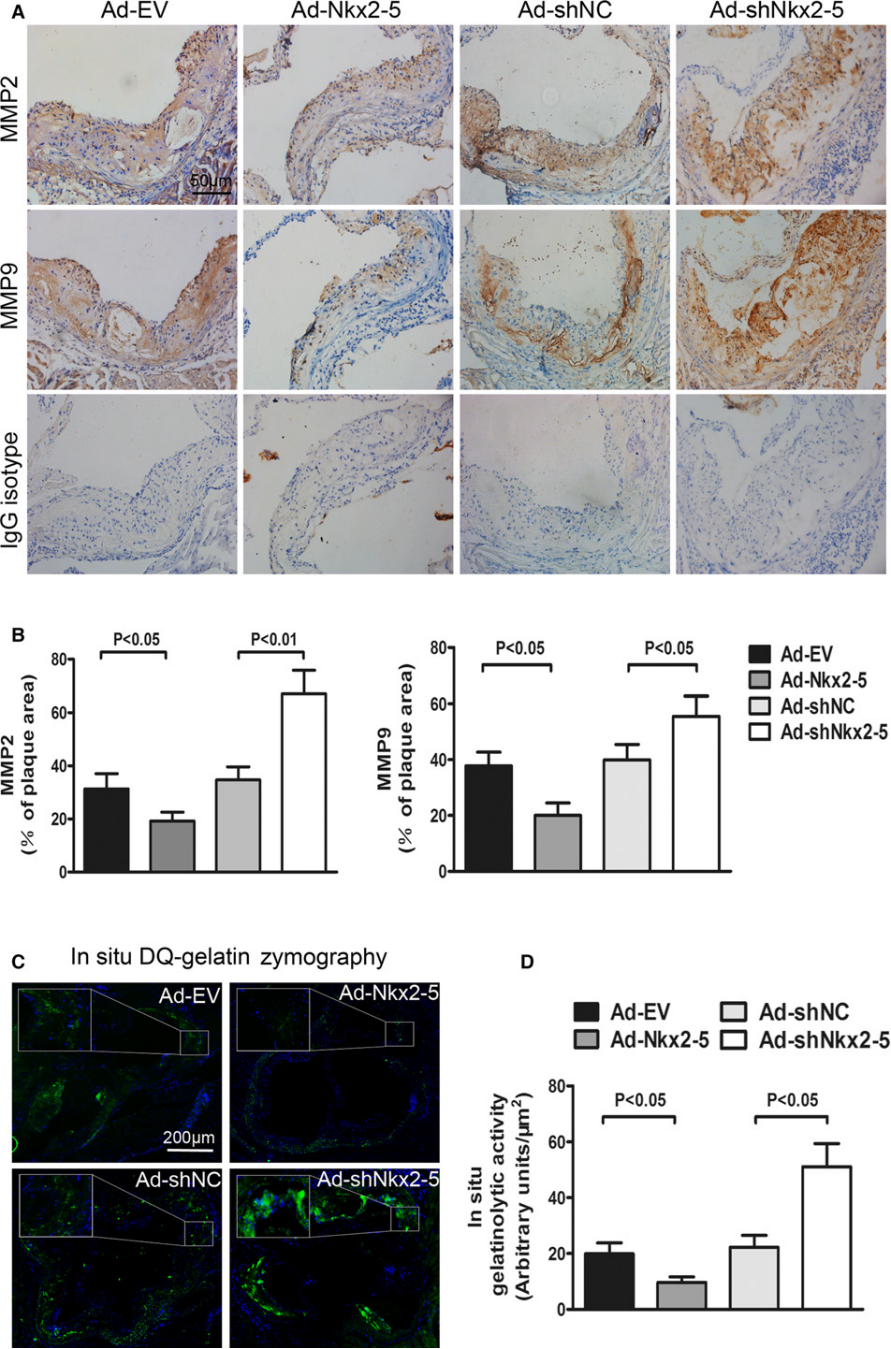
Effects of NK2 homeobox 5 (Nkx2‐5) on MMP2/9 expression and activities in ApoE^−/−^ mice fed a Western diet. A, Cross‐sections of aortic sinus were immunostained with antibodies against MMP2 and MMP9. Staining with rabbit IgG isotype was used as the negative control for MMP2 and MMP9. B, Quantification of histochemical staining of MMP2 and MMP9. Positive stained areas were quantified as a percentage of total plaque area. Data are expressed as mean±SEM (n=12 per group). C, In situ zymographic analysis of MMP2/9 activities in aortic sinus. Localization of green fluorescence indicates gelatinolytic activities of MMP2 and MMP9, and 4′,6‐diamidino‐2‐phenylindole (DAPI) was used for nucleus staining. All images were obtained under identical conditions of laser beam intensity and exposure time; representative images are displayed. D, Quantitative analysis of fluorescence intensity in (C). Data are expressed as mean±SEM (n=12 per group). DQ indicates dye‐quenched; IgG, immunoglobulin G; MMP, matrix metalloproteinase.

The MMPs are zinc‐dependent proteases that play a critical role in extracellular matrix degradation, tissue remodeling, aneurysm formation, and plaque rupture. We then examined whether plaque vulnerability was related to the action of MMPs. As immunohistochemical staining demonstrated, MMP2 and MMP9 expression in atherosclerotic lesions was substantially decreased in mice treated with Ad‐Nkx2‐5, compared to Ad‐EV‐treated controls, and Ad‐shNkx2‐5 infection significant increased the levels of MMP2 and MMP9 (Figure 4A and 4B). Given that the mere presence of MMP proteins may not establish their catalytic capacity, we further examined MMP enzymatic activity in atherosclerotic lesions using in situ fluorescent zymography. As illustrated in Figure 4C and 4D, obvious gelatinolytic activity, demonstrated as the presence of signal for gelatinolytic product formation, was observed in aortic sinus lesions. A relative weak signal was detected in mice treated with Ad‐Nkx2‐5, whereas a stronger gelatinolytic activity was observed in Ad‐shNkx2‐5‐treated mice.

### Nkx2‐5 Regulates the Expression of MMPs and TIMPs Directly in Human Monocyte‐Derived Macrophages and HASMCs

**Figure 5:**
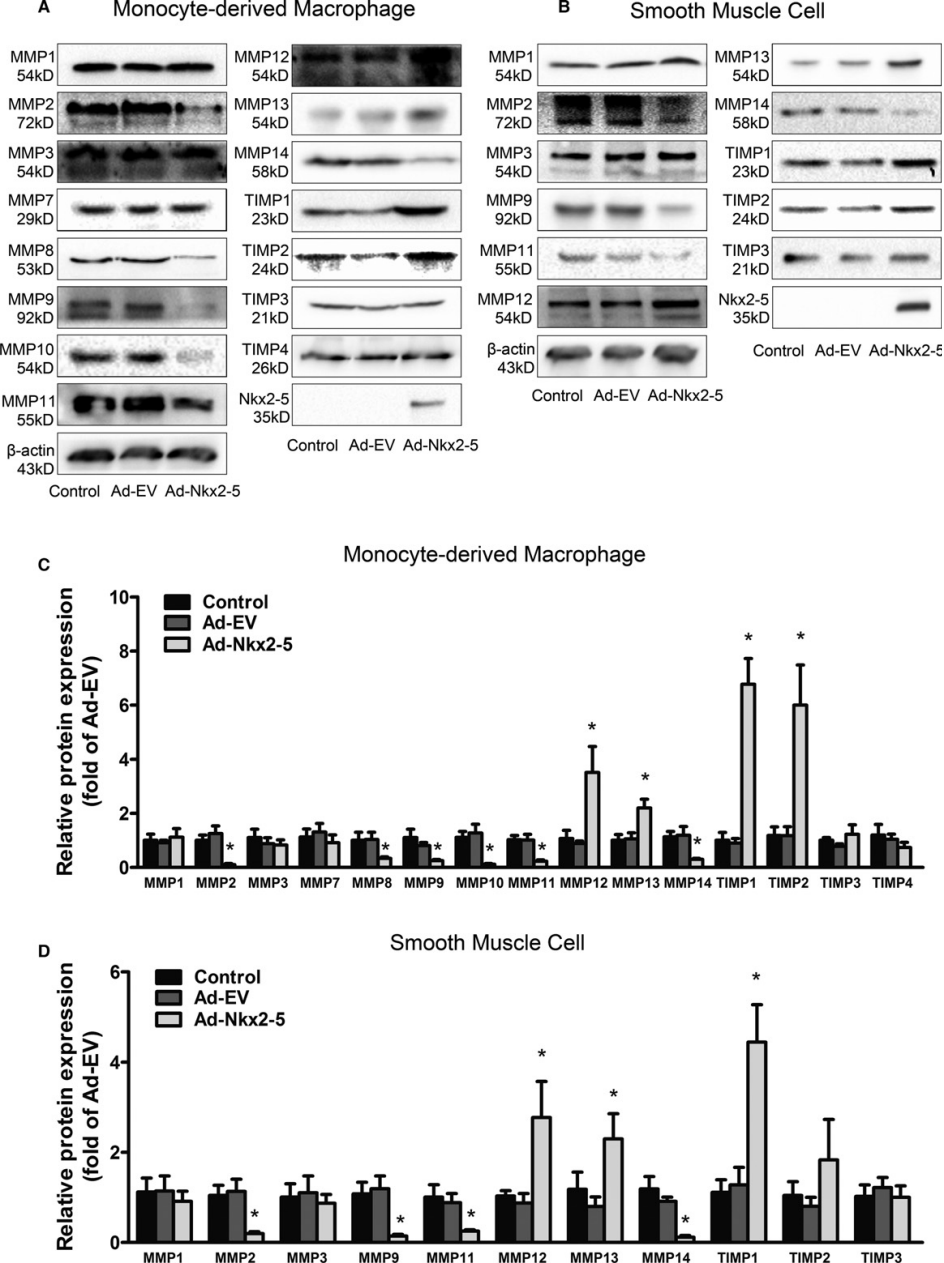
NK2 homeobox 5 (Nkx2‐5) regulates expression of MMPs and TIMPs in human monocyte‐derived macrophages and human aortic smooth muscle cells. A, Representative immunoblot for MMPs and TIMPs in human peripheral blood monocyte‐derived macrophages infected with Ad‐EV or Ad‐Nkx2‐5. B, Representative immunoblot for MMPs and TIMPs in human aortic smooth muscle cells infected with Ad‐EV or Ad‐Nkx2‐5. C, Quantification of band density in (A). Results are expressed as fold of control group. Data represent the mean±SEM of 3 independent experiments. *P<0.05 versus Ad‐EV group. D, Quantification of band density in (B). Results are expressed as fold of control group. Data represent the mean±SEM of 3 independent experiments. *P<0.05 versus Ad‐EV group. MMP indicates matrix metalloproteinase; TIMP, tissue inhibitor of metalloproteinase.

To examine whether expression of MMPs, including MMP2 and MMP9, was directly regulated by Nkx2‐5, protein levels of MMPs, as well as their main natural inhibitors, TIMPs, were quantified in human peripheral blood monocyte‐derived macrophages and HASMCs using western blot. Surprisingly, compared to the Ad‐EV‐treated group, MMP2, MMP8, MMP9, MMP10, MMP11, and MMP14 were all remarkably downregulated in macrophages infected with Ad‐Nkx2‐5, along with increased expression of MMP12 and MMP13, whereas the expression of MMP1, MMP3, and MMP7 were not significantly different. For the TIMP family, TIMP1 and TIMP2 were obviously induced by Ad‐Nkx2‐5 transfection, whereas expression of TIMP3 and TIMP4 were almost unaffected (Figure 5A and 5C). Moreover, in smooth muscle cells, the change tendency of MMPs and TIMPs was consistent with that observed in macrophages. Expression of MMP7, MMP8, MMP10, and TIMP4, however, were hardly detectable in smooth muscle cells, which was probably attributed to a very low protein level (Figure 5B and 5D).

**Figure 6:**
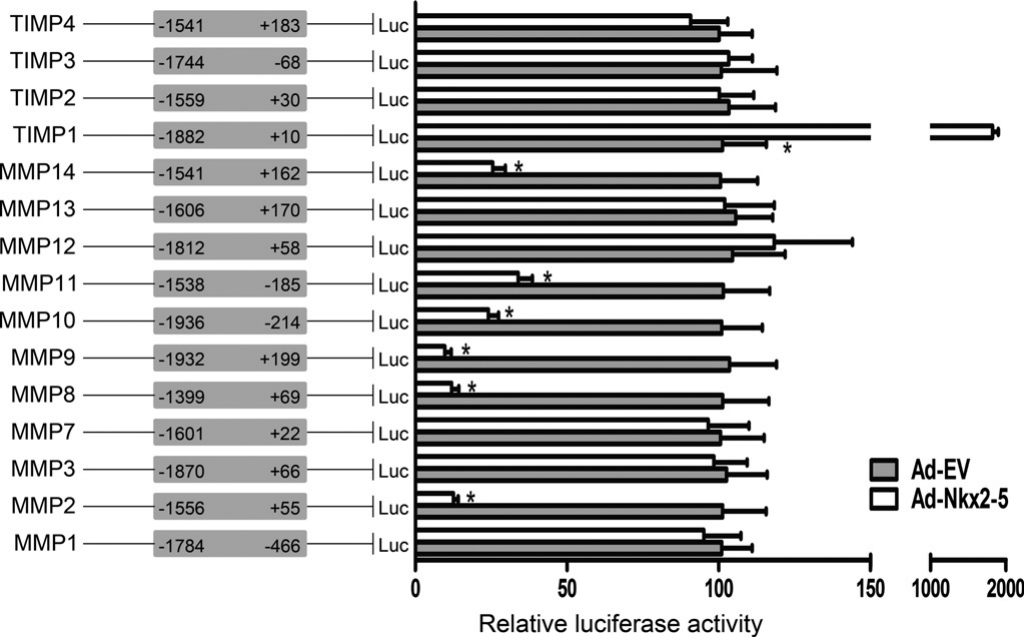
NK2 homeobox 5 (Nkx2‐5) directly regulates MMPs/TIMPs transcription. Luciferase (Luc) reporter constructs containing promoters of MMPs or TIMPs were cotransfected with an internal control plasmid pRL‐TK into HEK293 cells, followed by infection with Ad‐Nkx2‐5 or Ad‐EV. Schematic representation in the left panel demonstrates the range of promoters, and the numbers indicate nucleotide positions relative to the transcription initiation site. The right panel demonstrates relative luciferase activities, which are expressed as a percent of values determined in the Ad‐EV‐treated group. Data represent the mean±SEM of 3 independent experiments. *P<0.05 versus the Ad‐EV‐treated group. MMP indicates matrix metalloproteinase; TIMP, tissue inhibitor of metalloproteinase.

Nkx2‐5 was initially discovered as a cardiac homeobox transcription factor, which played a pivotal role in the transcriptional regulation of cardiac, vascular, and hematopoietic lineages. To explore whether Nkx2‐5 could also directly regulate transcription of MMPs and TIMPs, we established the promoter‐luciferase reporter constructs containing the sequences ranging from upstream 2000 base pairs (bp) to downstream 200 bp of gene transcription initiation sites. The dual luciferase reporter gene assay was conducted in HEK293 cells. As depicted in Figure 6, Nkx2‐5 overexpression significantly reduced the promoter activity of MMP2, MMP8, MMP9, MMP10, MMP11, and MMP14 and also enhanced TIMP1 promoter activity. The results indicated that the specific members of this family were most likely to be the novel target genes of Nkx2‐5.

To determine whether Nkx2‐5 showed high enrichment in promoters of MMPs/TIMPs, ChIP was performed in human monocytic leukemia cell line (THP1)‐derived macrophages. By bioinformatics analysis, several putative Nkx2‐5 binding sites, which contained the consensus sequence of 5′‐TNAAGTG‐3′ or 5′‐TTAATT‐3′, were found in each MMP or TIMP promoter ranging from −2000 to +200 bp, except for TIMP4, and they are denoted as “seq‐No.” in Figure S4. As expected, no significant occupancy of Nkx2‐5 on the promoters was observed in cells infected with Ad‐EV. However, for the Ad‐Nkx2‐5‐treated group, Nkx2‐5 enrichment in the specific binding sites of MMPs/TIMPs gene promoters was observed, including MMP2, MMP3, MMP8, MMP9, MMP10, MMP11, MMP14, and TIMP1 (Figure S4). The putative Nkx2‐5 binding sites of each promoter, as well as specific PCR primers and product lengths, are demonstrated in Table S3.

Besides MMP2 and MMP9, expression of MMP3, MMP8, MMP14, and TIMP1 was also examined in the atherosclerotic lesions of ApoE^−/−^ mice fed a Western diet. As expected, Nkx2‐5 gene transfer significantly inhibited expression of MMP8 and MMP14 and enhanced TIMP1 expression. There was no difference in the level of MMP3 between Ad‐Nkx2‐5‐treated mice and Ad‐EV‐treated mice (Figure S5).

### Nkx2‐5 Regulates Expression of Extra Functional Genes in Macrophages, Smooth Muscle Cells, and Endothelial Cells

**Figure 7:**
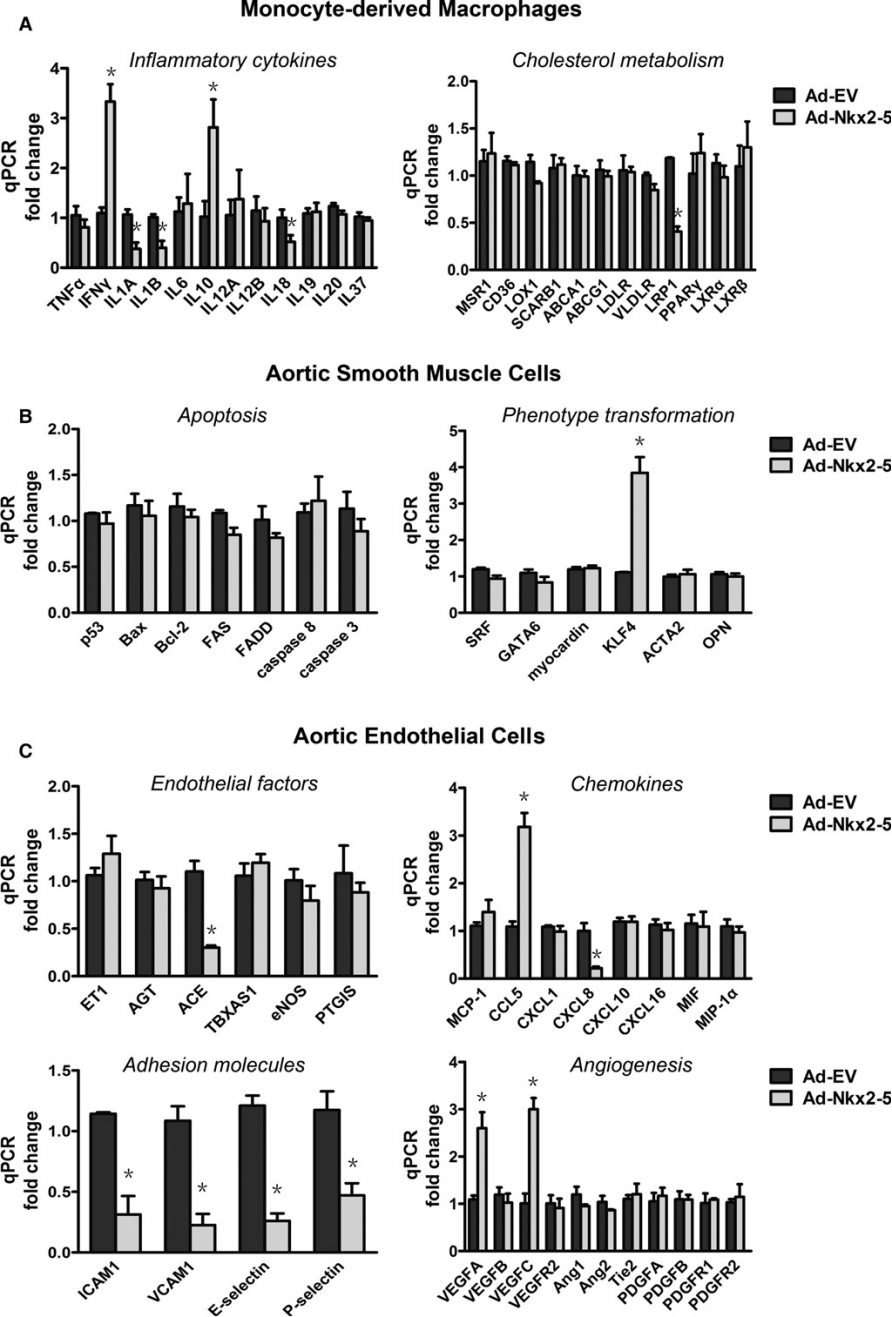
NK2 homeobox 5 (Nkx2‐5) regulates the expression of extra functional genes in macrophages, smooth muscle cells, and endothelial cells. Cells treated with Ad‐Nkx2‐5 or Ad‐EV for 24 hours were subjected to real‐time polymerase chain reaction analysis. A, Relative mRNA expression levels of genes involved in inflammation and cholesterol metabolism of monocyte‐derived macrophages. B, Relative mRNA expression levels of genes involved in apoptosis and phenotype transformation of aortic smooth muscle cells. C, Relative mRNA expression levels of endothelial factors involved in arterial vasomotoricity, chemokine production, adhesion, and angiogenesis. Data represent the mean±SEM of 3 independent experiments. *P<0.05 versus the Ad‐EV‐treated group. ICAM‐1 indicates intercellular adhesion molecule‐1; qPCR, quantitative polymerase chain reaction; VCAM‐1, vascular cell adhesion molecule‐1.

Atherosclerosis is a chronic vascular inflammatory disease, with a series of cytokine‐mediated interactions among multiple kinds of cells. Besides being involved in extracellular matrix remolding, whether Nkx2‐5 could exhibit far‐ranging effects on disease progression is still unknown. Expression of several key functional genes was detected in human peripheral blood monocyte‐derived macrophages, HASMCs, and HAECs. Reverse‐transcriptase PCR analysis demonstrated that macrophages infected with Ad‐Nkx2‐5 had significantly lower mRNA levels of the proinflammatory cytokines, interleukin (IL) 1α, IL1β, and IL18, along with upregulated IL10, a well‐known anti‐inflammatory cytokine. We could not ignore that Nkx2‐5 also augmented expression of interferon‐gamma, which could exacerbate atherosclerosis as an important activator of macrophages and inducer of adaptive immunity (Figure 7A). No appreciable changes of genes related to cholesterol metabolism and foam cell formation were observed (Figure 7A). Also, genes involved in apoptosis and phenotype transformation of smooth muscle cells were almost unaffected by Nkx2‐5 except for KLF4 (Figure 7B).

Moreover, whether Nkx2‐5 could be involved in regulation of endothelial functions was investigated (Figure 7C). Of note, in cultured aortic endothelial cells, Nkx2‐5 exhibited a potent inhibitory effect on mRNA levels of ICAM1, VCAM1, E‐selectin, and P‐selectin, each of which enhances leukocyte‐endothelium interactions and further promotes aggregation of macrophages in atherosclerotic lesions. The effects of Nkx2‐5 on chemokines production were confusing, given that expression of C‐X‐C motif chemokine ligand 8 was inhibited and C‐C motif chemokine ligand 5 augmented by Ad‐Nkx2‐5 infection. In addition, the local vascular renin‐angiotensin system and angiogenesis may also be affected, given that we observed that angiotensin‐converting enzyme (ACE) was inhibited and vascular endothelial growth factor (VEGF) A and C (VEGFA, VEGFC) upregulated.

### Nkx2‐5 Inhibits Monocyte‐Endothelial Adhesion and Decreases Expression of Adhesion Molecules in Early Atherosclerosis

**Figure 8:**
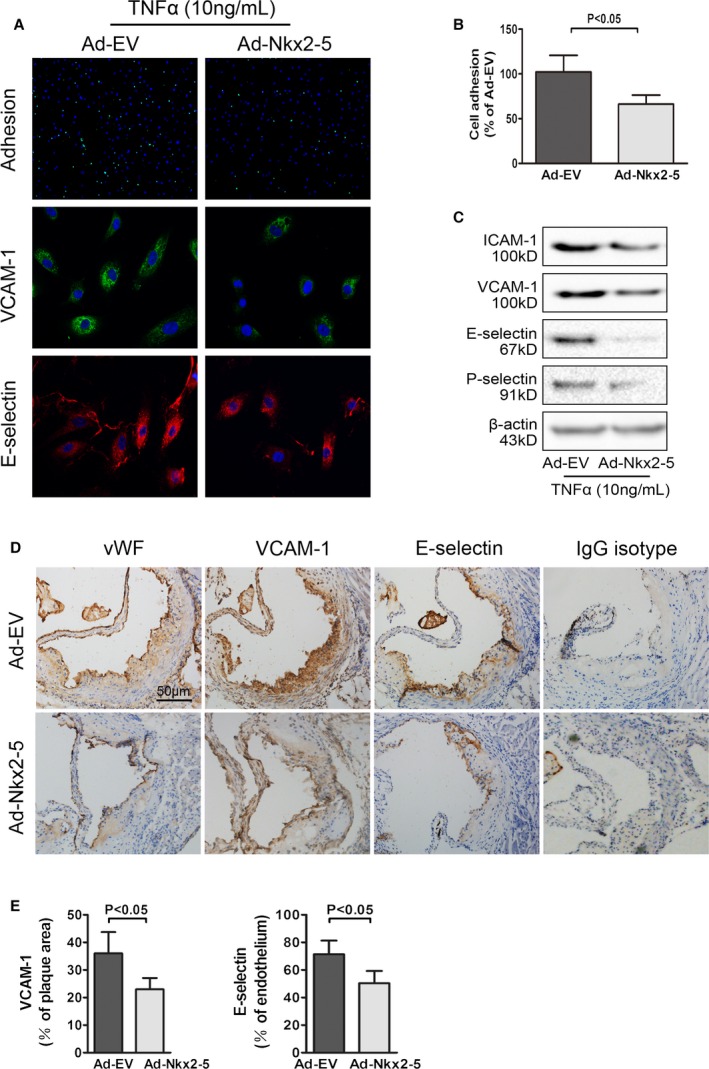
NK2 homeobox 5 (Nkx2‐5) inhibits monocyte‐endothelial adhesion and decreases expression of adhesion molecules in early atherosclerosis. A, Upper panel: representative images used for quantification of peripheral blood monocytes (PBMC; green, calcein AM) attached to aortic endothelial cells (HAECs, blue, DAPI). Middle panel: effects of Nkx2‐5 on expression of vascular cell adhesion molecule‐1 (VCAM‐1) in endothelial cells (representative immunofluorescence image: green for VCAM‐1 and blue for cell nuclei stained with DAPI). Lower panel: effects of Nkx2‐5 on expression of E‐selectin in endothelial cells (representative immunofluorescence image: red for E‐selectin and blue for cell nuclei stained with DAPI). B, Quantification of adhesion. The number of PBMC adhered to per 100 HAECs was calculated. Results are expressed as a percent of values determined in the Ad‐EV‐treated group. Data represent the mean±SEM of 3 independent experiments. C, Representative immunoblot for intercellular adhesion molecule‐1 (ICAM‐1), VCAM‐1, E‐selectin, and P‐selectin in endothelial cells infected with Ad‐EV or Ad‐Nkx2‐5. D, Cross‐sections of early atherosclerotic lesions in aortic sinus were immunostained with antibodies against von Willebrand factor (vWF), VCAM‐1, and E‐selectin. Staining with rabbit IgG isotype was used as the negative control. E, Quantification of histochemical staining of VCAM‐1 and E‐selectin. Positive stained areas were quantified as a percentage of total plaque area or plaque endothelium. Data are expressed as mean±SEM (n=12 per group). AM indicates acetomethoxy; DAPI, 4′,6‐diamidino‐2‐phenylindole; HAECs, human aortic endothelial cells; IgG, immunoglobulin G; TNFα, tumor necrosis factor alpha.

Given that compromised endothelial function is a hallmark of early atherosclerosis, the effects of Nkx2‐5 on cell adhesion were further characterized. An endothelial cell monolayer adhesion assay was conducted, and we determined that pretreatment of cultured aortic endothelial cells with Ad‐Nkx2‐5 could significantly reduce the number of peripheral blood monocytes attached to TNFα‐activated endothelial cell monolayers (Figure 8A and 8B). Simultaneously, expression of adhesion molecules ICAM1, VCAM1, E‐selectin, and P‐selectin were all decreased in endothelial cells, as evaluated by western blot and immunofluorescence assay (Figure 8A and 8C). In agreement with the above results, decreased expression of E‐selectin and VCAM1 were also found in early atherosclerotic lesions of Ad‐Nkx2‐5‐treated mice fed a chow diet, compared to their littermates treated with Ad‐EV. Positive stained areas were quantified as a percentage of total plaque area or plaque endothelium (Figure 8D and 8E).

Given that it has been reported that NF‐κB and MAPK signaling pathways play a critical role in mediating TNFα‐induced endothelial cell activation, we investigated whether Nkx2‐5 could suppress expression of adhesion molecules through inhibiting activation of these pathways. The three members of MAPK family, including c‐Jun N‐terminal kinase (JNK), extracellular signal‐regulated kinase (ERK), and p38, were activated at 5 minutes after TNFα stimulation, as well as NF‐κB pathway at 30 minutes (Figure S6A and S6C). However, Nkx2‐5 overexpression had no effects on MAPK and NF‐κB pathways in endothelial cells activated by TNFα or in the resting condition, as quantified by the relative phosphorylation of JNK, ERK, p38, inhibitor of NF‐κB, and p65 (Figure S6B and S6D).

Furthermore, the 2200‐bp promoter‐luciferase reporter constructs of ICAM1, VCAM1, E‐selectin, and P‐selectin were established and transfected into HEK293 cells. Nkx2‐5 overexpression significantly reduced the luciferase activity of all promoters except for VCAM1 (Figure S6E).

## Discussion

This study provides novel evidence in the antiatherogenic effects of Nkx2‐5, suggesting that Nkx2‐5 is a physiological protector against vascular diseases.

Rupture‐prone vulnerable plaques are susceptible to undergoing rapid progression and giving rise to superimposed thrombosis, ultimately leading to acute cardiovascular events. The vulnerable plaques are typically associated with the presence of highly inflammatory cell content and a large necrotic core covered by a thin, fibrous cap.^26^ Moreover, increased elastolytic activity in the arterial wall also accelerates the progression and rupture of atherosclerotic plaques.^27^ In contrast, the amount and organization of collagen and other matrix components is associated with the mechanical stability of the fibrous cap. In the present study, Nkx2‐5 not only decreased lesion areas, but also induced a highly characteristic architecture of more‐stable plaques, with decreased infiltrating macrophages, abundant collagen content, integral VSMC‐rich fibrous caps overlying small necrotic cores, and a relatively well‐preserved elastic lamina. It could be attributed, at least in part, to the regulation of Nkx2‐5 on MMPs and TIMPs. Molecular studies revealed that Nkx2‐5, as a key transcription factor, was recruited to the promoters of specific members of MMPs/TIMPs, thus directly regulated gene expression. As luciferase reporter gene assay demonstrated, Nkx2‐5 overexpression significantly reduced the promoter activity of MMP2, MMP8, MMP9, MMP10, MMP11, and MMP14 and also enhanced TIMP1 promoter activity. For other MMPs/TIMPs that were not modified by Nkx2‐5 in western blot assay (MMP1, MMP3, MMP7, TIMP3, and TIMP4), the promoter activities were unaffected by Nkx2‐5 as expected. The regulation mechanism was further clarified in ChIP assay; Nkx2‐5 enrichment in the specific binding sites of promoters was observed, including MMP2, MMP3, MMP8, MMP9, MMP10, MMP11, MMP14, and TIMP1. No significant occupancy of Nkx2‐5 on the promoters of MMP1, MMP7, TIMP3, and TIMP4 was observed. Although some inconsistencies could not be ignored, the luciferase activities of MMP12, MMP13, and TIMP2 promoters were not affected by Nkx2‐5, although western blot showed a significant increase in protein levels. This is probably because the promoter constructs, which range from −2000 to +200 bp, do not include the significant binding sites of Nkx2‐5. Alternatively, the elevated protein levels of MMP12 and MMP13 might be attributed to compensatory reduced protein degradation, given that the majority of MMPs, including MMP2, MMP8, MMP9, MMP10, MMP11, and MMP14, were all repressed by Nkx2‐5. Moreover, in ChIP assay, Nkx2‐5 showed high enrichment in the MMP3 promoter, yet it might make little sense, given that western blot or dual luciferase reporter gene assay did not demonstrate any significant differences.

Given that the intriguing association between the expression/activity of specific MMPs and signs of plaque inflammation and matrix degradation was depicted in the early 1990s, an impressive effort has been made to precisely define the roles of MMPs in both the development and complications of atherosclerosis.^28^, ^29^, ^30^ Overexpression of MMP1 in macrophages resulted in smaller aortic atherosclerotic lesions with reduced fibrillar collagen content in ApoE^−/−^ mice.^31^ Lesion areas were significantly larger in ApoE^−/−^MMP3^−/−^ mice than in ApoE^−/−^ mice.^32^ In contrast, MMP7 may not make a major contribution to atherosclerotic plaque development or stability.^33^ Mice lacking MMP13 accumulated more collagen with a more‐organized supramolecular structure in plaques; so did MMP14 deficiency. MMP14 was also related to the activity regulation of other MMPs, such as MMP2 and MMP13.^34^, ^35^, ^36^, ^37^ Deficiency of MMP2, MMP8, or MMP9 led to smaller atherosclerotic lesions with fewer smooth muscle cells and reduced macrophage infiltration.^38^, ^39^, ^40^, ^41^, ^42^ The roles of MMP12 in atherosclerosis are controversial and studies on MMP10 and MMP11 are still limited. Given that the majority of MMPs could be regulated by Nkx2‐5, it was hard to determine which dominated in the progression of atherosclerosis and a comprehensive effect should be taken into consideration.

Compromised endothelial function is a hallmark of early atherosclerosis, and the leukocyte‐endothelium interaction constitutes a key cellular event in the initiation of the disease.^43^ Although we failed to detect Nkx2‐5 expression in the endothelium of atherosclerotic plaques, Nkx2‐5 gene transfer clearly ameliorated atherosclerosis at an early stage. It would be owing to the inhibitory effects on the expression of adhesion molecules such as E‐selectin, P‐selectin, ICAM1, and VCAM1. Activated NF‐κB and MAPK pathways, which certainly augmented the expression of these adhesion molecules, could not be inhibited by Nkx2‐5 either in resting conditions or in TNFα‐activated cells. In contrast, the promoter luciferase activities of E‐selectin, P‐selectin, and ICAM1 were obviously suppressed by Nkx2‐5. It indicated that these adhesion molecules, similar to MMPs and TIMPs, would most likely be the novel target genes of Nkx2‐5. The luciferase activity of VCAM1 promoter was unaffected, probably because the key regulation region was not included in the construct, given that western blot and immunofluorescence in endothelial cells, as well as immunohistochemistry in atherosclerotic lesions, all demonstrated a significant decrease in VCAM1 protein level.

Besides involving in extracellular matrix remodeling and leukocyte‐endothelium interaction, Nkx2‐5 could exert more actions predicted to influence the progression of atherosclerosis, such as inflammatory response, phenotypic switching, and arterial vasomotoricity. Krüppel‐like factor 4 (KLF4), which is not expressed in adult‐differentiated smooth muscle cells, was upregulated by Nkx2‐5 in vitro. Several studies provide evidence that KLF4 is re‐expressed and involved in mediating smooth muscle cell phenotypic switching within lesions of ApoE^−/−^ mice on a Western diet as well as following vascular injury.^44^, ^45^ Key questions include the following. Is Nkx2‐5 involved in the increased expression of KLF4 in atherosclerotic lesions? Is KLF4‐dependent smooth muscle cell phenotypic switching beneficial or detrimental? Considering a variety of target genes of Nkx2‐5 involved in atherosclerosis, how much of a difference will KLF4 make? More research is needed to elaborate these questions. Nkx2‐5 also exerted an inhibitory effect on ACE, a key enzyme of renin‐angiotensin‐aldosterone system (RAAS). Accumulating evidence suggests that inappropriate activation of local tissue RAAS in vascular tissue, immune cells, and perivascular adipose tissue is associated with endothelial dysfunction and arterial stiffness.^46^, ^47^, ^48^ However, a conclusion could not be reached simply because the activity of this enzyme has not been determinated. This holds for some other critical enzymes, such as endothelia nitric oxide (NO) synthase, thromboxane A synthase 1, and prostaglandin I2 synthase. Further analysis of enzyme activities will help us clarify the exact role of Nkx2‐5 in the endothelial functions. Moreover, upregulation of VEGFA and VEGFC in Ad‐Nkx2‐5‐treated endothelial cells seemed inconsistent with our previous conclusion, given that VEGF induces endothelial cell proliferation, promotes cell migration, and increases cell adhesion.^49^, ^50^, ^51^ However, the effects of VEGF may not predominate given that leukocyte‐endothelial cell adhesion was obviously inhibited by Nkx2‐5. There are reasons to think that these effects were attributed to the direct inhibition of Nkx2‐5 on major adhesion molecules. Furthermore, roles of VEGF in vascular biology remain highly controversial. Both systemic and local VEGF gene transfer protected from neointimal growth, a phenomenon that has been reported to be, in part, NO dependent.^52^, ^53^, ^54^ Concomitantly, systemic VEGF inhibition accelerates experimental atherosclerosis and disrupts endothelial homeostasis.^55^ A single intraperitoneal application of VEGF, however, did increase atherosclerosis in different animal models.^56^ The opposing effects of VEGF appear to markedly depend on local VEGF concentrations, stage of the disease, and different experimental conditions. Although these additional functions were just preliminarily validated in cultured cells, we have reason to believe that the effects of Nkx2‐5 on atherosclerosis are comprehensive, involving multiple kinds of cells and different pathological processes.

In the present study, intraventricular adenovirus gene transfer was used to manipulate Nkx2‐5 expression in aorta, and exposure of mice to a Western diet before adenovirus administration reflected the situation of elderly patients with pre‐existing atherosclerosis. However, whether the antiatherogenic effects of Nkx2‐5 were partially mediated by cross‐talk between arteries and remote organs remained elusive, given that systemic adenovirus gene transfer actually affected various tissues and dynamic multiple organ cross‐talk in atherosclerosis has been paid much emphasis.^57^, ^58^ To further elucidate the effects of Nkx2‐5 on specific cell types of certain tissues, cell‐type–selective genetic manipulation should be taken into account in our future studies.

Taken together, our present study suggests that Nkx2‐5 could ameliorate and stabilize the advanced atherosclerotic plaques, which may partly be attributed to the regulation on MMPs and TIMPs in macrophages and smooth muscle cells. As well, Nkx2‐5 significantly represses expression of adhesion molecules in endothelial cells and therefore improves endothelium functions and inhibits progression of atherosclerosis at an early stage.

## Sources of Funding

This work was supported by the National Natural Science Foundation of China (30971245 to Kai Huang and 81000112 to Dan Huang).

## Disclosures

None.

## Supporting information


**Table S1.** qRT‐PCR Primers Used
**Table S2.** Primers Designed for Promoter Reporter Gene Construction
**Table S3.** Specific Primers Used in Chromatin Immunoprecipitation Assay
**Table S4.** Plasma Lipid Profiles and Body Weights of Adenovirus Treated ApoE^−/−^ Mice Fed Standard Chow Diet or Western Diet
**Figure S1.** Nkx2‐5 expression in aortic sinus and aorta of diseased ApoE^−/−^ and normal C57BL/6 mice.
**Figure S2.** Adenovirus infection efficiency inarterial walls of experimental atherosclerotic mice was examined by western blot.
**Figure S3.** Lymphocyte subtypes were not affected by Nkx2‐5 gene transfer.
**Figure S4.** Chromatin immunoprecipitation assay of Nkx2‐5 interaction with the promoters of MMPs and TIMPs.
**Figure S5.** Effects of Nkx2‐5 on MMP3, MMP8, MMP14, and TIMP1 expression in ApoE^−/−^ mice fed a Western diet.
**Figure S6.** Nkx2‐5 regulates expression of adhesion molecules at the transcriptional level in endothelial cells.
