# Supplementary material for: Nkx2‐5 Is Expressed in Atherosclerotic Plaques and Attenuates Development of Atherosclerosis in Apolipoprotein E–Deficient Mice
Source: J Am Heart Assoc. 2016 Dec 19;5(12):e004440. doi: 10.1161/JAHA.116.004440 (PMC5210424; doi:10.1161/JAHA.116.004440)
Supplement: Supplementary file 1 — Table S1. qRT‐PCR Primers Used Table S2. Primers Designed for Promoter Reporter Gene Construction Table S3. Specific Primers Used in Chromatin Immunoprecipitation Assay Table S4. Plasma Lipid Profiles and Body Weights of Adenovirus Treated ApoE−/− Mice Fed Standard Chow Diet or Western Diet Figure S1. Nkx2‐5 expression in aortic sinus and aorta of diseased ApoE−/− and normal C57BL/6 mice. Figure S2. Adenovirus infection efficiency inarterial walls of experimental atherosclerotic mice was examined by western blot. Figure S3. Lymphocyte subtypes were not affected by Nkx2‐5 gene transfer. Figure S4. Chromatin immunoprecipitation assay of Nkx2‐5 interaction with the promoters of MMPs and TIMPs. Figure S5. Effects of Nkx2‐5 on MMP3, MMP8, MMP14, and TIMP1 expression in ApoE−/− mice fed a Western diet. Figure S6. Nkx2‐5 regulates expression of adhesion molecules at the transcriptional level in endothelial cells. [file JAH3-5-e004440-s001.pdf]

SUPPLEMENTAL MATERIAL

Table S1. qRT-PCR primers used

Gene	Forward primer	Reverse primer
TNFα	CCCAGGGACCTCTCTCTAATC	ATGGGCTACAGGCTTGTCACT
IFNγ	TCGGTAACTGACTTGAATGTCCA	TCGCTTCCCTGTTTTAGCTGC
IL1A	TGGTAGTAGCAACCAACGGGA	ACTTTGATTGAGGGCGTCATTC
IL1B	TTCGACACATGGGATAACGAGG	TTTTTGCTGTGAGTCCCGGAG
IL6	ACTCACCTCTTCAGAACGAATTG	CCATCTTTGGAAGGTTCAGGTTG
IL10	GACTTTAAGGGTTACCTGGGTTG	TCACATGCGCCTTGATGTCTG
IL12A	ATGGCCCTGTGCCTTAGTAGT	AGCTTTGCATTCATGGTCTTGA
IL12B	ACCCTGACCATCCAAGTCAAA	TTGGCCTCGCATCTTAGAAAG
IL18	TCTTCATTGACCAAGGAAATCGG	TCCGGGGTGCATTATCTCTAC
IL19	ATCCAAGCTAAGGACACCTTCC	GTCACGCAGCACACATCTAAG
IL20	ATGAAAGCCTCTAGTCTTGCCT	GCCCCGTATCTCAGAAAATCC
IL37	TTCTTTGCATTAGCCTCATCCTT	CGTGCTGATTCCTTTTGGGC
MSR1	GCAGTGGGATCACTTTCACAA	AGCTGTCATTGAGCGAGCATC
CD36	GGCTGTGACCGGAACTGTG	AGGTCTCCAACTGGCATTAGAA
LOX1	AATGATAGAAACCCTTGC	TTCCCAGTTAAATGAGCC
SCARB1	AATAAGCCCATGACCCTGAAGC	GCCCCACATGATCTCACCC
ABCA1	ACCCACCCTATGAACAACATGA	GAGTCGGGTAACGGAAACAGG
ABCG1	ATTCAGGGACCTTTCCTATTCGG	CTCACCACTATTGAACTTCCCG
LDLR	ACGGCGTCTCTTCCTATGACA	CCCTTGGTATCCGCAACAGA
VLDLR	AGAAAAGCCAAATGTGAACCCT	CACTGCCGTCAACACAGTCT

LRP1	CTTCAAGAACGCAGTGGTG	CAGTAGAGTTTGCTTTCAGGGA
PPARγ	ACCAAAGTGCAATCAAAGTGGA	ATGAGGGAGTTGGAAGGCTCT
LXRα	CCTTCAGAACCCACAGAGATCC	ACGCTGCATAGCTCGTTCC
LXRβ	AGAAGATTCGGAAACAACAGCA	GCTGGATCATTAGTTCTTGAGCC
p53	CCACCATCCACTACAACTACAT	AGGACAGGCACAAACACG
Bax	TCTGACGGCAACTTCAACTG	AGGAAAACGCATTATAGACCAC
Bcl-2	CTGGGAGAACAGGGTACGATAA	GGCTGGGAGGAGAAGATGC
FAS	GATGGCCAATTCTGCCATAAG	GTCTGGTTCATCCCCATTGACT
FADD	GTGGCTGACCTGGTACAAGAG	GGTAGATGCGTCTGAGTTCCAT
caspase 3	CATGGAAGCGAATCAATGGACT	CTGTACCAGACCGAGATGTCA
caspase 8	GTTGTGTGGGGTAATGACAATCT	TCAAAGGTCGTGGTCAAAGCC
SRF	CCGGCAAGGCACTGATTCA	CTCATTCTCTGGTCTGTTGTGG
GATA6	CTCAGTTCCTACGCTTCGCAT	GTCGAGGTCAGTGAACAGCA
myocardin	CCACCTATGGACTCAGCCTAC	CTCAGTGGCGTTGAAGAAGAG
KLF4	CCCACATGAAGCGACTTCCC	CAGGTCCAGGAGATCGTTGAA
ACTA2	AAAAGACAGCTACGTGGGTGA	GCCATGTTCTATCGGGTACTTC
OPN	GAAGTTTCGCAGACCTGACAT	GTATGCACCATTCAACTCCTCG
ET1	AGAGTGTGTCTACTTCTGCCA	CTTCCAAGTCCATACGGAACAA
AGT	CCCCAGTCTGAGATGGCTC	GACGAGGTGGAAGGGGTGTA
ACE	GGAGGAATATGACCGGACATCC	TGGTTGGCTATTTGCATGTTCTT
TBXAS1	GTATGGACCTCTGTGTGGGTA	CCGACGCCATTCTGTTGGTAA
eNOS	TGATGGCGAAGCGAGTGAAG	ACTCATCCATACACAGGACCC

PTGIS	CTGTTGGGCGATGCTACAGAA	GCCTCAATTCCGTAAAGAGTCA
MCP-1	CTTCTGTGCCTGCTGCTCAT	CGGAGTTTGGGTTTGCTTGTC
CCL5	CCAGCAGTCGTCTTTGTCAC	CTCTGGGTTGGCACACACTT
CXCL1	CCCCAAGAACATCCAAAGTG	GATGCAGGATTGAGGCAAG
CXCL8	GTCCTTGTTCCACTGTGCCT	GCTTCCACATGTCCTCACAA
CXCL10	GTGGCATTCAAGGAGTACCTC	TGATGGCCTTCGATTCTGGATT
CXCL16	CCCGCCATCGGTTCAGTTC	CCCCGAGTAAGCATGTCCAC
MIF	GAACCGCTCCTACAGCAAGCT	GCGAAGGTGGAGTTGTTCCA
MIP-1α	AGTTCTCTGCATCACTTGCTG	CGGCTTCGCTTGGTTAGGAA
ICAM1	TTGGGCATAGAGACCCCGTT	GCACATTGCTCAGTTCATACACC
VCAM1	GGGAAGATGGTCGTGATCCTT	TCTGGGGTGGTCTCGATTTTA
E-selectin	GCACAGCCTTGTCCAACC	ACCTCACCAAACCCTTCG
P-selectin	ATGGGTGGGAACCAAAAAGG	GGCTGACGGACTCTTGATGTAT
VEGFA	AGGGCAGAATCATCACGAAGT	AGGGTCTCGATTGGATGGCA
VEGFB	GAGATGTCCCTGGAAGAACACA	GAGTGGGATGGGTGATGTCAG
VEGFC	GAGGAGCAGTTACGGTCTGTG	TCCTTTCCTTAGCTGACACTTGT
VEGFR2	GGCCCAATAATCAGAGTGGCA	CCAGTGTCATTTCCGATCACTTT
Ang1	AGCGCCGAAGTCCAGAAAAC	TACTCTCACGACAGTTGCCAT
Ang2	CTCGAATACGATGACTCGGTG	TCATTAGCCACTGAGTGTTGTTT
Tie2	TTAGCCAGCTTAGTTCTCTGTGG	AGCATCAGATACAAGAGGTAGGG
PDGFA	GCAAGACCAGGACGGTCATTT	GGCACTTGACACTGCTCGT
PDGFB	CTCGATCCGCTCCTTTGATGA	CGTTGGTGCGGTCTATGAG

PDGFR1	TTTTTGTGACGGTCTTGGAAGT	TGTCTGAGTGTGGTTGTAATAGC
PDGFR2	TGATGCCGAGGAACTATTCATCT	TTTCTTCTCGTGCAGTGTCAC

Table S2. Primers designed for promoter reporter gene construction

Gene	Primers	Promoter region (bp)	Promoter length (bp)
**MMP1**	**F** ATCTGCCACTCCTTGACT	-1784 ～ -466	1319
	**R** GCTTCCCTTAATCTTTACTC		
**MMP2**	**F** CAAGACATAATCGTGACCTCCAAT	-1556 ～ +55	1611
	**R** ACCGCCTGAGGAAGTCTGGAT		
**MMP3**	**F** TATTATCTATCAGGCTTTCC	-1870 ～ +66	1936
	**R** TTTCCACTGGCTTTACTT		
**MMP7**	**F** TAGGATTACAGGCGTGAG	-1601 ～ +22	1623
	**R** TTGGACCTATGGTTGATTTG		
**MMP8**	**F** TACTCTAGCACCCATCAC	-1399 ～ +69	1468
	**R** CTTTCTTTCTGTCCCTCT		
**MMP9**	**F** GCCCAAGGTCACATAGCT	-1932 ～ +199	2051
	**R** CTCCAGGGAAGAGCACAA		
**MMP10**	**F** ATGGCAGCACAGTAGGTT	-1936 ～ -214	1723
	**R** ATTGGCTAGATAATTCACGT		
**MMP11**	**F** AGGCCCTTCATCATTTCAG	-1538 ～ -185	1354
	**R** GGCAAAGTCCCTATCTGGT		
**MMP12**	**F** AATGGAGTAGCCTGTAAT	-1812 ～ +58	1870
	**R** CTTTCTAGCCTAAGTTCC		
**MMP13**	**F** CTCTAAGGCACTGGCTAC	-1616 ～ +170	1776

	**R** AGGATTGGCAAGATACTC		
**MMP14**	**F** TAGGCCATGTACAGCTGGCATC	-1541 ～ +162	1703
	**R** ACCGCTGTCGGCTTGGAGTT		
**TIMP1**	**F** TCACCCGAGGTCAGGAGT	-1882 ～ +10	1892
	**R** GCCGACGAAAGGAGATACAC		
**TIMP2**	**F** CGCCTCTCGGGTTCAAGCGA	-1559 ～ +30	1589
	**R** CGCGCTGCCTTCTACGGATG		
**TIMP3**	**F** TATTAGGCTCATGGACACC	-1744 ～ -68	1677
	**R** AGAAAGGCAAGAGGAAGT		
**TIMP4**	**F** CTACACTGAGGTAGCCATAC	-1541 ～ +183	1724
	**R** GAAAGAGCCACAAAGACA		
**ICAM1**	**F** CTGCCCTGTCATCTCCCT	-1753 ～ -86	1668
	**R** TCCATTTCACAAAGCGGTA		
**VCAM1**	**F** CTGGGAGGAGCAGGTAGGA	-1718 ～ +66	1784
	**R** TTGTTGCAGAGGCGGAGG		
**E-selectin**	**F** TTTGGGTCTTGACATCTT	-1903 ～ +67	1970
	**R** GGTATCACTGCTGCCTCT		
**P-selectin**	**F** AGCGTGATAGGTATTGTT	-1822 ～ +61	1883
	**R** TCTGTGACTCTGCTGGTT		

Table S3. Specific primers used in Chromatin immunoprecipitation Assay

Gene	Putative binding site of Nkx2-5	Primers	Product length (bp)
**MMP1**	**seq1** GGGCTCAAGTGATTCCCCT	**F** ACCTCAGCCTCTTCAGTG	194
	(-559 ～ -541)	**R** GCTCAAGCCTATAATCCC	
	**seq2** TAAAGTGAGTGCTGGGGGA	**F** GGCAGCTTAACAAAGGCAGAA	144
	(-387 ～ -369)	**R** CAGGGCAGAGGGTGGAAT	
**MMP2**	**seq1** AGACATAATCGTGACCTCC	**F** CTGAAGCCCACTGAGACC	141
	(-1555 ～ -1536)	**R** GCAGGGAACAGTTTGAGAA	
	**seq2** AAGAGTGAGTGGGGAATTC	**F** GCTGAAGTCAGGCGTTCC	214
	(-1015 ～ -999)	**R** AGCCCTCAGTTCCACGAA	
**MMP3**	**seq1** AGGAGAATCACTTGAGCCC	**F** CTGCCACCACTCTGTTCT	211
	(-1499 ～ -1481)	**R** TGGGCTCAAGTGATTCTCC	
	**seq2** TCCAATGTTTATTAAGAAA	**F** TCTTCAGTCATAGGGATC	162
	(-1264 ～ -1246)	**R** AGACCTTTAATAAGTGCC	
	**seq3** TGTTCAGGTAATTAACACT	**F** AGACAACATAGAGCTAAG	168
	(+164～ +182)	**R** CCACCTGGCCAGGTCAGT	
**MMP7**	**seq1** TGCTCATTCACTTGAGGAA	**F** TTTCCCAGGCTTGTCTCA	200
	(-1523 ～ -1505)	**R** TTTTGGTAATATTTGCAT	
	**seq2** AAAGAAAACACTCAAATGA	**F** CTGCCAATAACGATGTAA	132
	(-83 ～ -65)	**R** CTTCTCAGCCTCGAATGT	

	**seq3** AGGCATGAGTGAGCTACAG	**F** AGCTATGCGACTCACCGTG	157
	(+122～ +140)	**R** AGATGGCAAAAGAATGGAA	
**MMP8**	**seq1** AGACTCAAGTGGGAGACTA	**F** CCTTGTCTTCTGCCTGTG	187
	(-825 ～ -807)	**R** TGTTTCATTTGTGGAGGG	
	**seq2** AATCATAATTTTTTAGCAA	**F** ACAAATGTCTGGGCAATC	145
	(-583 ～ -565)	**R** CTTCTGGAGGATGTGGTT	
	**seq3** TAAGTTAATTCAACCTCAA	**F** CCACATACAATGAGGGAG	172
	(-303 ～ -285)	**R** AAAGGAACAAGGGACTAA	
	**seq4** AGCTGTGAGTGACACATGA	**F** CCTATGTTGCTTCATATT	204
	(-10 ～ +9)	**R** GAAATGGAAGCGTCTTCAGG	
**MMP9**	**seq1** GAAGTTAATTATCTCCATC	**F** AATCCAGGACTTCGTGAC	151
	(-1617 ～ -1598)	**R** TAAAGGGCCTACTATGTG	
	**seq2** GGGCAGATCACTTGAGTCA	**F** CCCGTAATCCTAGCACTT	257
	(-1474 ～ -1455)	**R** TGTAGTATCACTCTGTCACCC	
	**seq3** TGTGATAATTGGGGCTGGA	**F** TTTCCCAGGCTTGTCTCA	201
	(-1072 ～ -1053)	**R** TTTTGGTAATATTTGCAT	
**MMP10**	**seq1** ATTATCAAGAATTATGTAC	**F** AGTCATTTATCACTATTAT	231
	(-1985 ～ -1967)	**R** AAGTTACCCAGTCTCAGG	
	**seq2** GAACATAATTATACACTGG	**F** AGCAAAGAAGAGGAAGAGGGTA	209
	(-613 ～ -595)	**R** TACTGGCCGTGGAGTAGGG	
	**seq3** CTACTTAATTCTTACCTGC	**F** CCACATTTAGACCACGAC	65
	(-365 ～ -347)	**R** AAGCAGGTAAGAATTAAGTAGG	

	**seq4** TCCTCTGAGTGGGGCAGCA	**F** CCAGTAGACAAAGAAGGTA	126
	(+91 ～ +109)	**R** TCCTTGTTGGAGTCCTCCT	
**MMP11**	**seq1** GGGTTCAAGTGATTCTCCT	**F** AGTCTCGCTCTGTCACCC	119
	(-1330 ～ -1311)	**R** CCTGTAAGCCCAGCTACTC	
	**seq2** CAGTACTCCACTCAGACAC	**F** TTCTTTCAAGTCTGAGGTGGCT	105
	(-1034 ～ -1015)	**R** TGGGAGGTGTCTGAGTGGAGTA	
	**seq3** GGGCAGATCACTTGAGGTC	**F** TTCCCTATCTGTAAACTTCGG	173
	(-745 ～ -726)	**R** GACCTCAAGTGATCTGCCCACC	
	**seq4** TGCAGTGAGTGGAGATCAC	**F** GCGGAGGTTGCAGTGAGTGG	119
	(-578 ～ -559)	**R** CCCTGTGAGGAAAGGGATA	
**MMP12**	**seq1** TTAAGCCTCACTCAAGCAG	**F** ATCACAGAAGGCAAGTCT	284
	(-1006 ～ -988)	**R** ATTGCTCTTACCCACCTC	
	**seq2** TTAGAAAGCACTCATTTAC	**F** GATCTGAGCCGGTACAAC	242
	(-382 ～ -364)	**R** CTTAAAGCCTGATATTCTTG	
	**seq3** TTGCTTGAGTGATGGACTA	**F** GGATAGGTGGACGTAGAGG	195
	(-130 ～ -112)	**R** AGTCCGGGTTCTGTGAAT	
**MMP13**	**seq1** CTCTTAGTCACTCAAATTA	**F** CTGGCTACTCTAGATTATA	177
	(-1263 ～ -1245)	**R** CTTTAAATAAATGATTGG	
	**seq2** ACCTTCAAGTGACTGGGAA	**F** AGTCGCCACGTAAGCATG	220
	(-90 ～ -72)	**R** TCACCACCACTGGGAAGG	
**MMP14**	**seq1** TGACCTAATTTCTGTCCAC	**F** CTTCTATTCTTCTGCCACA	180
	(-1035 ～ -1016)	**R** GACTATCAGCGAAATCTAA	

	**seq2** GCGAGTTCCAATTAAAGGT (-591 ～ -572)	**F** AGTCCCAACTCCCAATCC<br>**R** TCTGTGGCTCCAACCTTT	178
	**seq3** AATCAAGCCACTCAGAATA (-243 ～ -224)	**F** CCAATAATTCCCACCCTG<br>**R** ACACCTCTAAGTTGCCTTTT	189
**TIMP1**	**seq1** TACTGACCCACTCACTTGC (-833 ～ -814)	**F** GGAAGGTTCCTGCATTGTC<br>**R** AAAGAAGCAAGTGAGTGGGT	142
	**seq2** AGCTTTGAGTGAGATAAAC (-576 ～ -557)	**F** AAAACGGGAATAAGAACC<br>**R** GAGTCAATACATGGCAGAAC	189
	**seq3** TATGCTGAGTGCCTGGTAT ***** (-557 ～ -538)	**F** AAAACGGGAATAAGAACC<br>**R** GAGTCAATACATGGCAGAAC	189
**TIMP2**	**seq1** GGCCTCAAGTGATCCTCCT (-1096 ～ -1078)	**F** GGCCTCAAGTGATCCTCCT<br>**R** AGAGGGAAGCCCAGGAAA	197
	**seq2** TGACCTGAGTGCAGCAGTG (-937 ～ -919)	**F** CGCCCTGATTCTTCCTGT<br>**R** GCTGTGAACCTGCCTGTG	186
**TIMP3**	**seq1** TGCTTTCATAATTAAGTAG (-1421 ～ -1402)	**F** GTAATTTCGTTACTTTTCAG<br>**R** AGCAGCAGTTTTCTCATA	113

***** The "seq3" of TIMP2 could not be distinguished from "seq2" by specific primers since the two binding sites get close to each other

Table S4. Plasma lipid profiles and body weights of adenovirus treated ApoE$^{-/-}$ mice fed standard chow diet or western diet.

	**Standard Chow Diet (20 weeks)**			
	**Ad-EV**	**Ad-Nkx2-5**	**Ad-EV**	**Ad-shNkx2-5**
Total cholesterol (mg/dl)	272.3±36.6	331.1±60.9	306.6±24.2	277.4±58.2
Triglycerides (mg/dl)	64.1±9.2	58.3.2±9.2	55.9±5.2	60.6±8.5
LDL cholesterol (mg/dl)	331.1.4±55.2	301.0±40.4	322.4.4±35.2	349.7±68.9
HDL cholesterol (mg/dl)	45.3±6.1	41.4±7.9	48.3±5.2	46.7±6.0
Body weight (g)	33.1±4.5	31.9+2.8	32.8±6.4	34.1±6.2
	**Western Diet (from 6 weeks to 22 weeks)**			
	**Ad-EV**	**Ad-Nkx2-5**	**Ad-EV**	**Ad-shNkx2-5**
Total cholesterol (mg/dl)	1213.2±105.7	1157.5±92.4	1202.6±155.1	1277±163.1
Triglycerides (mg/dl)	125.7±19.9	118.2±15.5	120.6±9.8	122.4±16.9
LDL cholesterol (mg/dl)	882.4±95.1	901.2±107.8	910.4±88.2	892.5±101.2
HDL cholesterol (mg/dl)	40.1±6.2	44.3±5.7	41.7±3.9	42.4±5.1
Body weight (g)	29.7±2.2	30.9+2.8	31.5±2.4	30.2±3.7

LDL, low-density lipoprotein; HDL, high-density lipoprotein

Figure S1.Nkx2-5 expression in aortic sinus and aorta of diseased ApoE$^{-/-}$ and normal C57BL/6 mice.

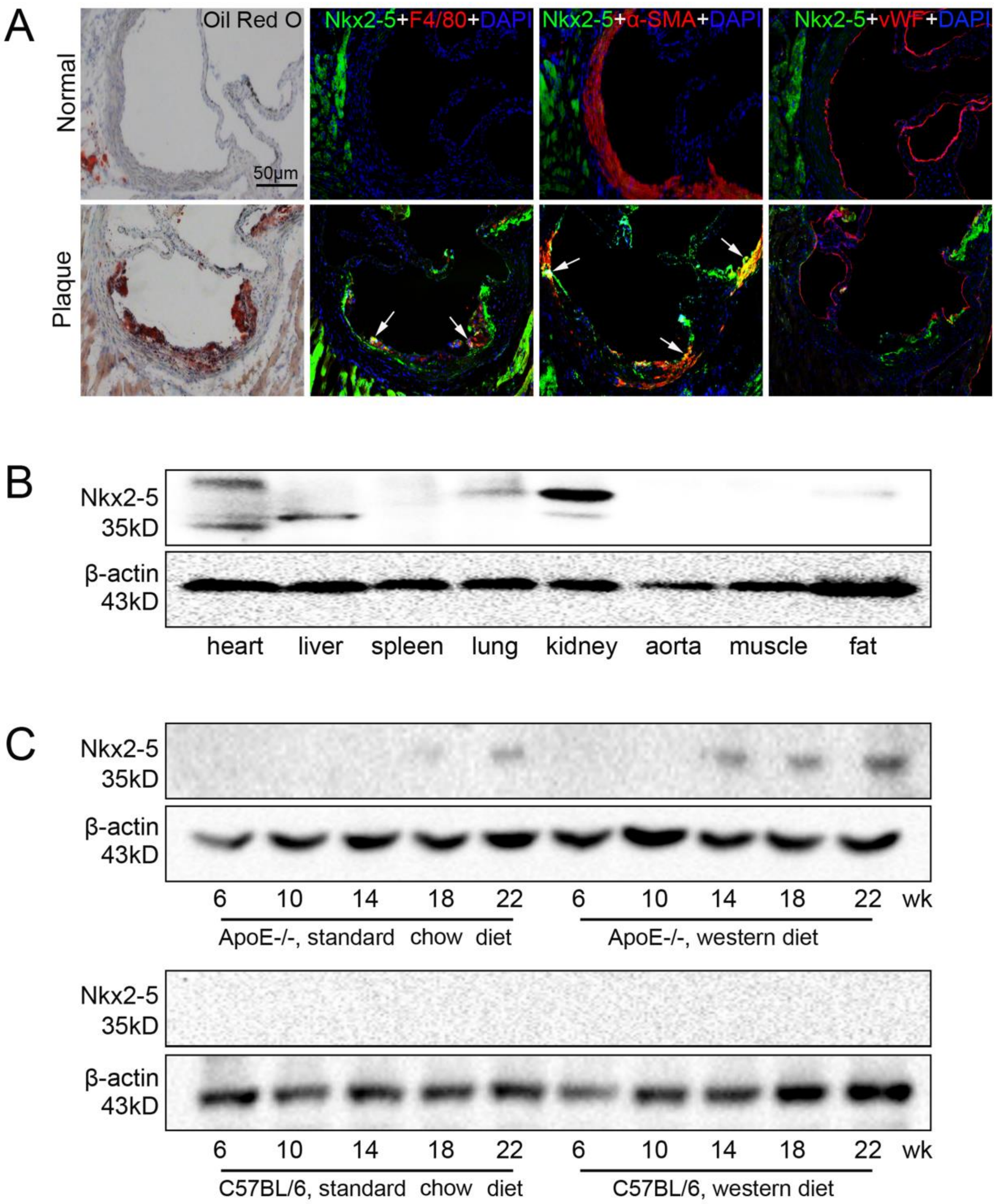

A. Immunofluorescence assay of Nkx2-5 in aortic sinus of C57BL/6 mice (normal, upper) and ApoE$^{-/-}$ mice (plaque, lower). For colocalization analysis, sections were co-stained for Nkx2-5

(green) and F4/80 (red, macrophage marker), α-SMA (red, smooth muscle cell marker) or von Willebrand Factor (red, vWF, endothelial marker). 4',6-diamidino-2-phenylindole (DAPI) was used for nucleus staining (blue). Arrows indicate Nkx2-5 and cell specific marker double positive cells. n=4 for C57BL/6 and 5 for ApoE$^{-/-}$ mice. **B.** Expression and distribution of Nkx2-5 in C57BL/6 mice. **C.** The temporal expression pattern of Nkx2-5 in the aorta of ApoE$^{-/-}$ and C57BL/6 mice, fed with standard chow diet or western diet respectively. n=6 for each group.

Figure S2. Adenovirus infection efficiency in arterial walls of experimental atherosclerotic mice was examined by Western blot.

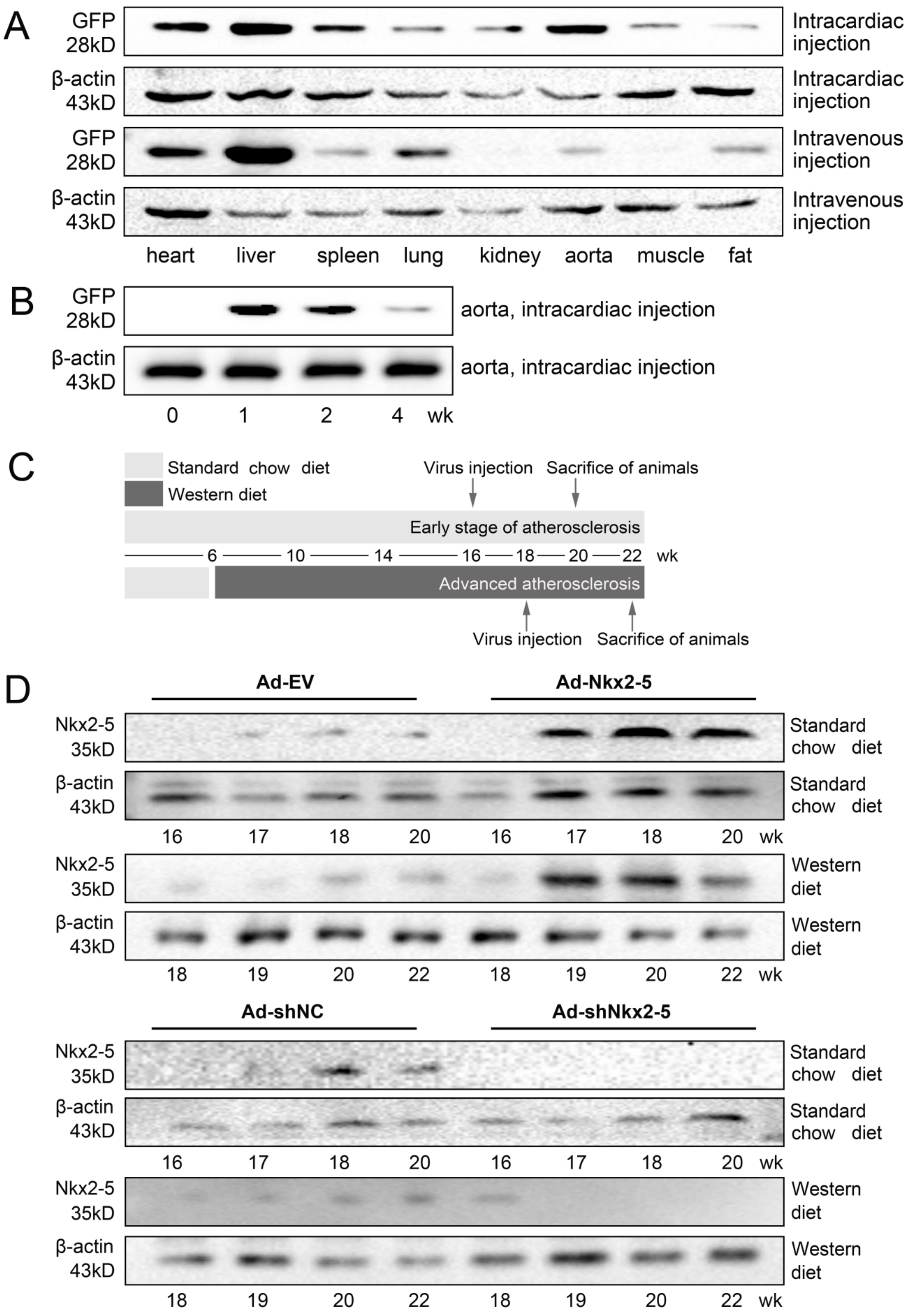
A
GFP 28kD
Intracardiac injection
β-actin 43kD
Intracardiac injection
GFP 28kD
Intravenous injection
β-actin 43kD
Intravenous injection
heart liver spleen lung kidney aorta muscle fat
B
GFP 28kD
aorta, intracardiac injection
β-actin 43kD
aorta, intracardiac injection
0 1 2 4 wk
C
Standard chow diet
Western diet
Virus injection
Sacrifice of animals
Early stage of atherosclerosis
6 10 14 16 18 20 22 wk
Advanced atherosclerosis
Virus injection
Sacrifice of animals
D
Ad-EV
Ad-Nkx2-5
Nkx2-5 35kD
Standard chow diet
β-actin 43kD
Standard chow diet
16 17 18 20 16 17 18 20 wk
Nkx2-5 35kD
Western diet
β-actin 43kD
Western diet
18 19 20 22 18 19 20 22 wk
Ad-shNC
Ad-shNkx2-5
Nkx2-5 35kD
Standard chow diet
β-actin 43kD
Standard chow diet
16 17 18 20 16 17 18 20 wk
Nkx2-5 35kD
Western diet
β-actin 43kD
Western diet
18 19 20 22 18 19 20 22 wk

A. Expression of GFP in various tissues of mice at 1 week after virus administration intraventricularly or intravenously. **B.** Levels of GFP expression in aortic tissue at 1, 2, or 4 weeks after virus administration intraventricularly. **C.** Experimental design. Two types of atherosclerotic lesions (referred to as early and advanced stage) were created in $ApoE^{-/-}$ mice through 2 different dietary manipulations. Four weeks before sacrificed, adenovirus was delivered by intraventricular injection. **D.** Expression levels of Nkx2-5 in aorta of mice treated with either Ad-EV, Ad-Nkx2-5, Ad-shNC or Ad-shNkx2-5 at indicated time points.

Figure S3. The lymphocytes subtypes were not affected by Nkx2-5 gene transfer.

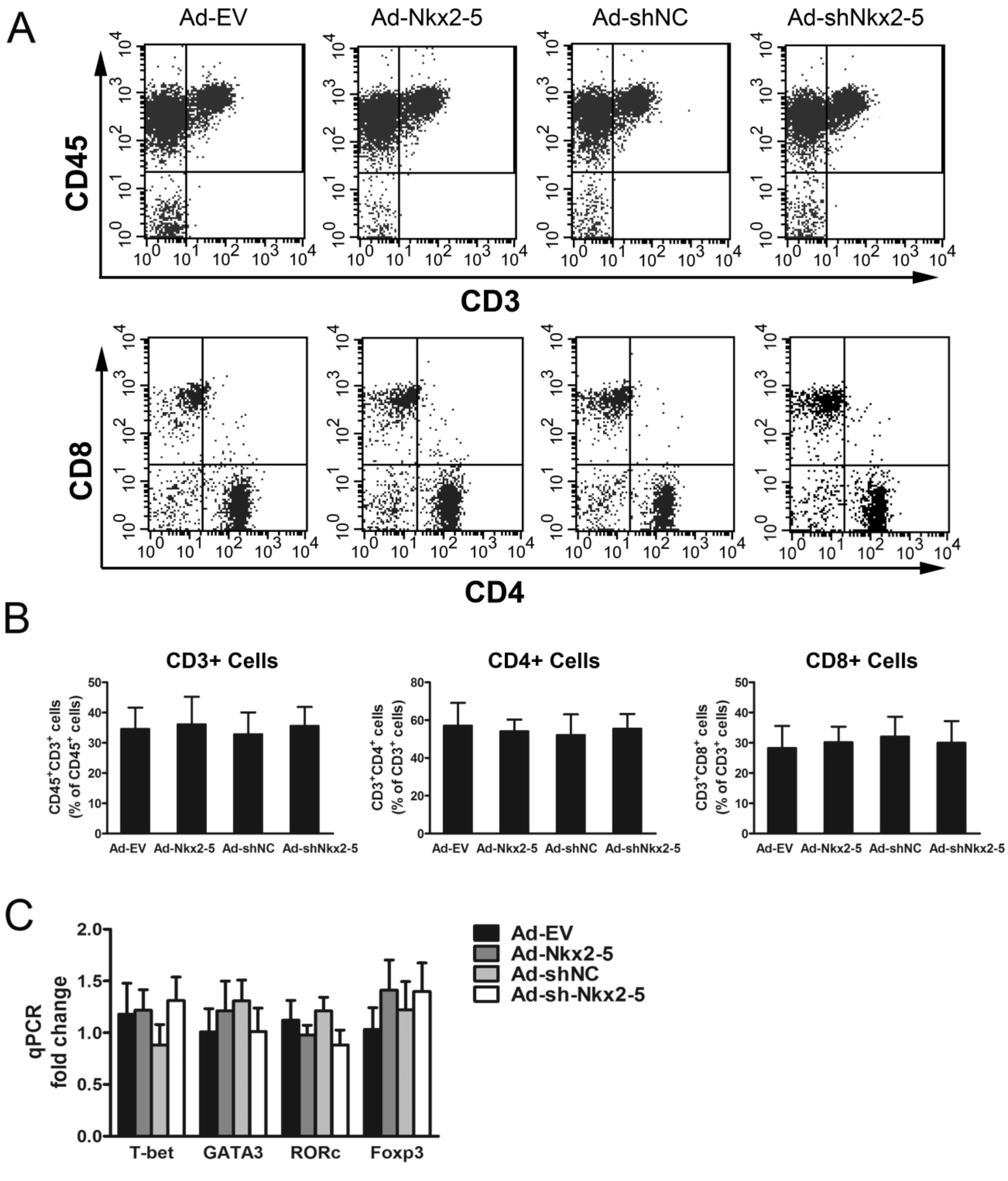

A. The profile of $CD3^+$, $CD4^+$ and $CD8^+$ cells were determined by flow cytometry in the spleen of Western diet fed $ApoE^{-/-}$ mice treated with Ad-EV, Ad-Nkx2-5, Ad-shNC or Ad-shNkx2-5. **B.**

Quantitative analysis of % $CD3^+$ cells, % $CD4^+$ cells and % $CD8^+$ cells in panel **A**. Data are expressed as mean ± SEM (n=8 for Ad-EV, 7 for Ad-Nkx2-5, 8 for Ad-NC and 10 for Ad-shNkx2-5 treated mice). **C.** Relative mRNA expression of transcription factors representative for T-cell subsets is measured in the spleen of Western diet fed $ApoE^{-/-}$ mice treated with Ad-EV, Ad-Nkx2-5, Ad-shNC or Ad-shNkx2-5. Data are expressed as mean ± SEM (n=8 per group).

Figure S4. Chromatin immunoprecipitation assay of Nkx2-5 interaction with the promoters of MMPs and TIMPs.

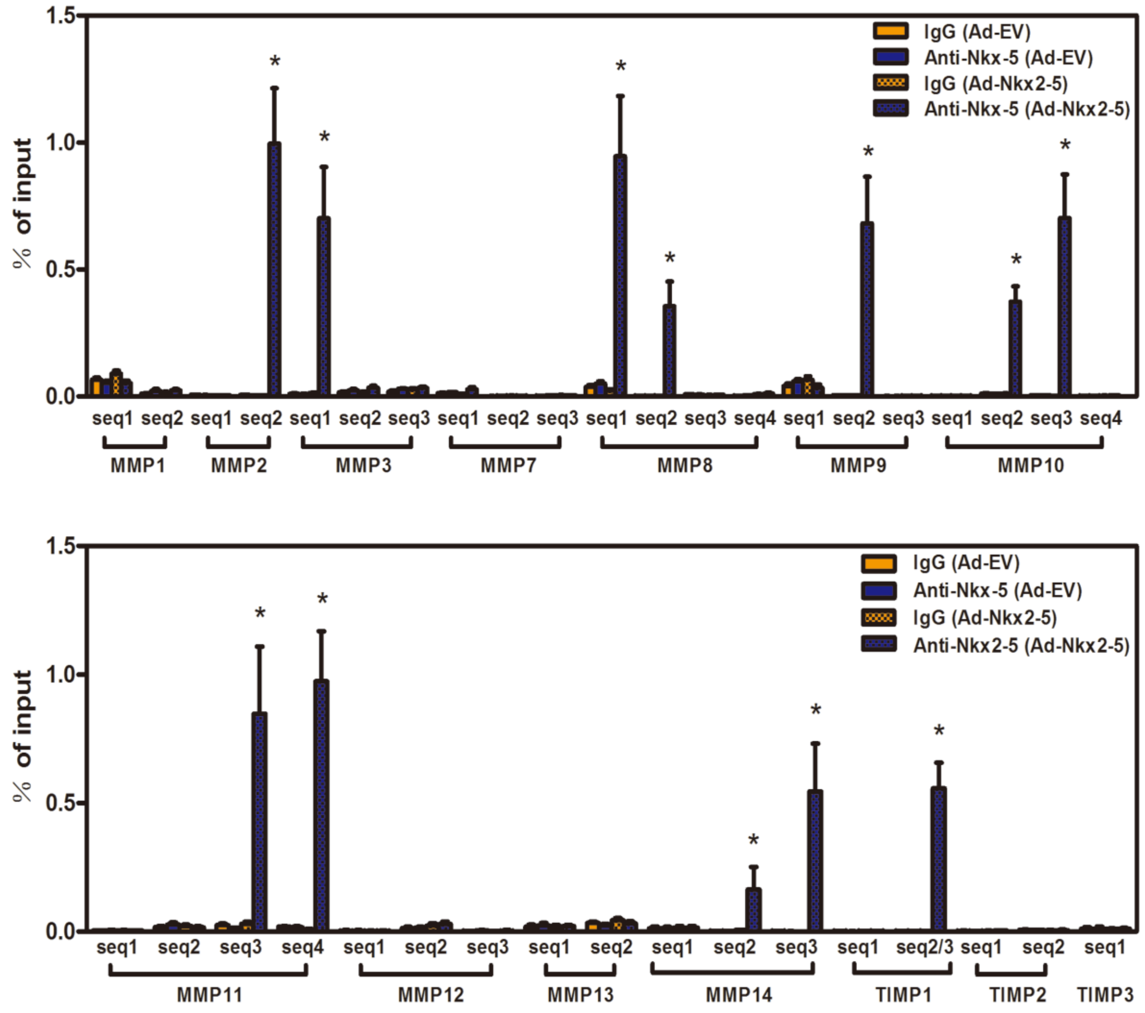

Chromatin immunoprecipitation assay was performed in human monocytic leukemia cell line (THP1) derived macrophages. IgG was used as a negative control. The putative Nkx2-5 binding sites of each MMP or TIMP promoter were denoted as “seq-No.”. Values are expressed as percent of amplified signals from IP chromatin to amplified input signals obtained from the same sample. Data are expressed as mean±SEM of three independent experiments. * $P < 0.05$ vs. IgG (Ad-Nkx2-5) group.

Figure S5. Effects of Nkx2-5 on MMP3, MMP8, MMP14 and TIMP1 expression in ApoE$^{-/-}$ mice fed western diet.

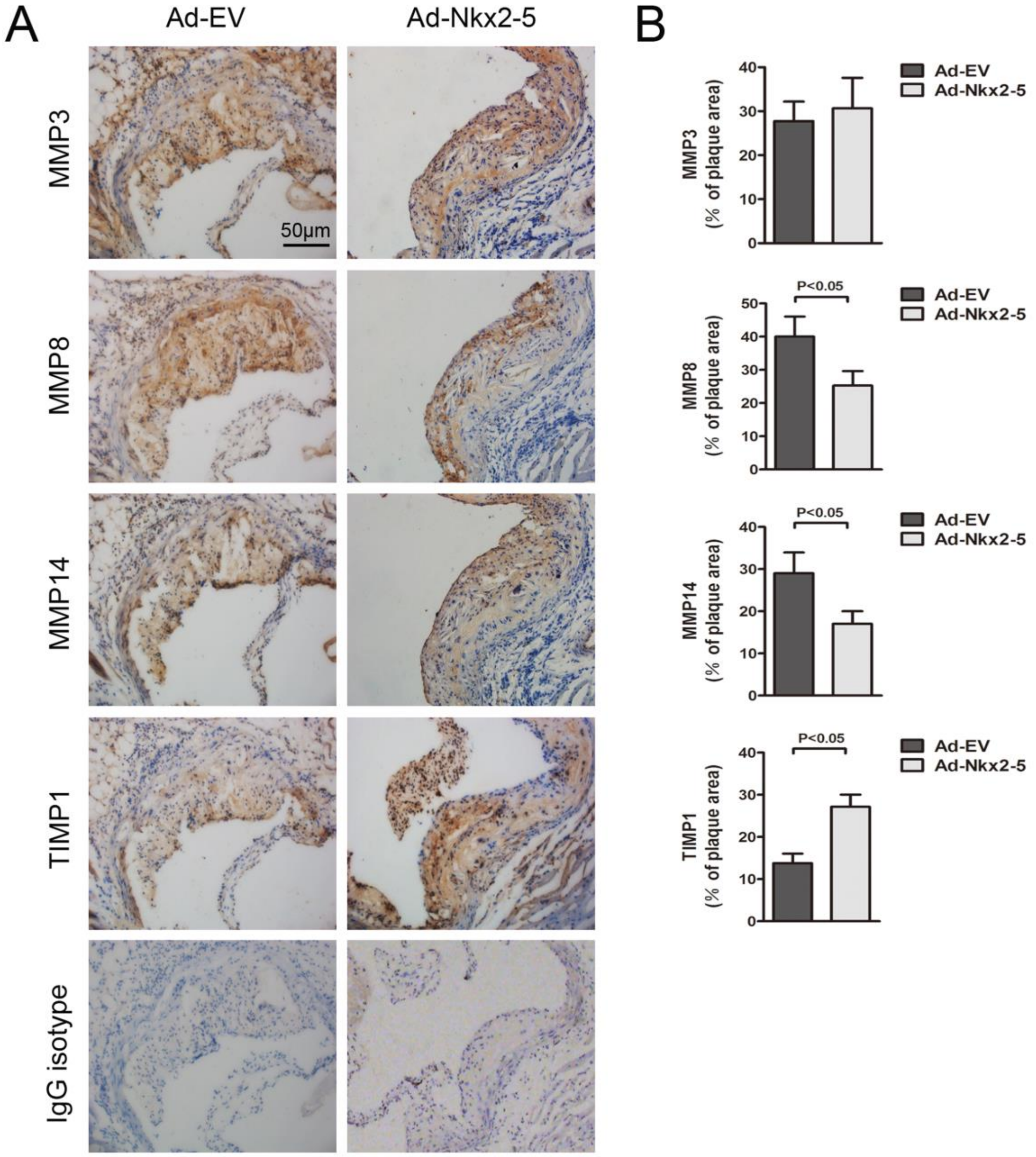

A. Cross-sections of aortic sinus were immunostained with antibodies against MMP3, MMP8, MMP14 and TIMP1. Staining with rabbit IgG isotype was used as the negative control. **B.** Quantification of the histochemical staining of MMP3, MMP8, MMP14 and TIMP1. Positive stained areas were quantified as a percentage of total plaque area. Data are expressed as mean±SEM (n=12 per group).

Figure S6. Nkx2-5 regulates the expression of adhesion molecules at transcriptional level in endothelial cells.

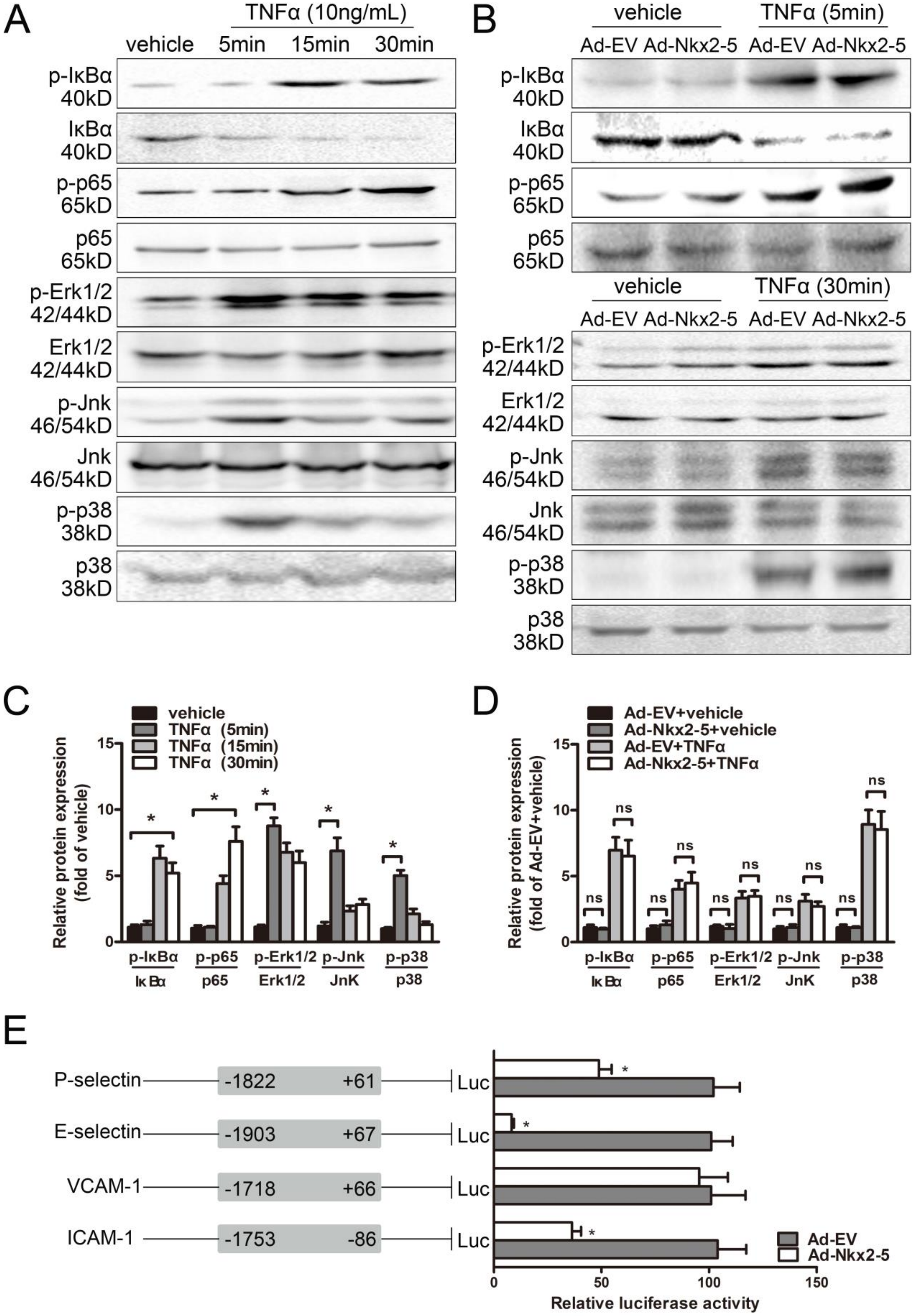
A
TNFα (10ng/mL)
vehicle
5min
15min
30min
p-IκBα 40kD
IκBα 40kD
p-p65 65kD
p65 65kD
p-Erk1/2 42/44kD
Erk1/2 42/44kD
p-Jnk 46/54kD
Jnk 46/54kD
p-p38 38kD
p38 38kD
B
vehicle
TNFα (5min)
Ad-EV Ad-Nkx2-5 Ad-EV Ad-Nkx2-5
vehicle
TNFα (30min)
Ad-EV Ad-Nkx2-5 Ad-EV Ad-Nkx2-5
C
vehicle
TNFα (5min)
TNFα (15min)
TNFα (30min)
Relative protein expression (fold of vehicle)
p-IκBα / IκBα
p-p65 / p65
p-Erk1/2 / Erk1/2
p-Jnk / JnK
p-p38 / p38
D
Ad-EV+vehicle
Ad-Nkx2-5+vehicle
Ad-EV+TNFα
Ad-Nkx2-5+TNFα
Relative protein expression (fold of Ad-EV+vehicle)
ns
p-IκBα / IκBα
p-p65 / p65
p-Erk1/2 / Erk1/2
p-Jnk / JnK
p-p38 / p38
E
P-selectin -1822 +61 Luc
E-selectin -1903 +67 Luc
VCAM-1 -1718 +66 Luc
ICAM-1 -1753 -86 Luc
Ad-EV
Ad-Nkx2-5
Relative luciferase activity

A. Human aortic endothelial cells were stimulated with TNFα (10ng/mL) for 5min, 10min and 30min. Western blot shows the phosphorylation levels of nuclear factor-kappaB (NF-κB) and mitogen-activated protein kinase (MAPK) signaling pathways at indicated time points. **B.** The effects of Nkx2-5 on the phosphorylation levels of NF-κB and MAPK pathways were examined by Western blot, either in the resting endothelial cells or TNFα-activated endothelial cells (5min for MAPK pathway and 30min for NF-κB pathway). **C.** Quantification of band density in **A**. Results are expressed as fold of vehicle group. Data represent the mean±SEM of three independent experiments. * $P < 0.05$ vs. vehicle group. **D.** Quantification of band density in **B**. Results are expressed as fold of Ad-EV+vehicle group. Data represent the mean±SEM of three independent experiments. *ns*, no significance. **E.** Luciferase reporter constructs containing promoters of ICAM-1, VCAM-1, E-selectin and P-selectin were co-transfected with an internal control plasmid pRL-TK into HEK293 cells, followed by infection with Ad-Nkx2-5 or Ad-EV. Schematic representation in the left panel demonstrates the range of promoters, and the numbers indicate nucleotide positions relative to the transcription initiation site. The right panel demonstrates the relative luciferase activities, which are expressed as a percent of values determined in Ad-EV treated group. Data represent the mean±SEM of three independent experiments. * $P < 0.05$ vs. Ad-EV treated group.
